# Microbiome Diversity and Community-Level Change Points within Manure-based small Biogas Plants

**DOI:** 10.3390/microorganisms8081169

**Published:** 2020-08-01

**Authors:** Susanne Theuerl, Johanna Klang, Benedikt Hülsemann, Torsten Mächtig, Julia Hassa

**Affiliations:** 1 Department Bioengineering, Leibniz Institute for Agricultural Engineering and Bioeconomy, Max-Eyth-Allee 100, 14469 Potsdam, Germany; jklang@atb-potsdam.de (J.K.); or jhassa@CeBiTec.Uni-Bielefeld.de (J.H.); 2 University of Hohenheim, The State Institute of Agricultural Engineering and Bioenergy, 70599 Stuttgart, Germany; Benedikt.Huelsemann@uni-hohenheim.de; 3 Kiel University, Institute of Agricultural Engineering, 24098 Kiel, Germany; tstefan@ilv.uni-kiel.de; 4 Center for Biotechnology (CeBiTec), Genome Research of Industrial Microorganisms, Bielefeld University, 33615 Bielefeld, Germany

**Keywords:** manure-based small biogas plants, microbial diversity, dynamic variations, 16S rRNA gene amplicon sequencing, terminal restriction fragment length polymorphism (TRFLP), nonmetric multidimensional scaling (NMDS), Threshold Indicator Taxa Analysis (TITAN), plant performance

## Abstract

Efforts to integrate biogas plants into bioeconomy concepts will lead to an expansion of manure-based (small) biogas plants, while their operation is challenging due to critical characteristics of some types of livestock manure. For a better process understanding, in this study, three manure-based small biogas plants were investigated with emphasis on microbiome diversity. Due to varying digester types, feedstocks, and process conditions, 16S rRNA gene amplicon sequencing showed differences in the taxonomic composition. Dynamic variations of each investigated biogas plant microbiome over time were analyzed by terminal restriction fragment length polymorphism (TRFLP), whereby nonmetric multidimensional scaling (NMDS) revealed two well-running systems, one of them with a high share of chicken manure, and one unstable system. By using Threshold Indicator Taxa Analysis (TITAN), community-level change points at ammonium and ammonia concentrations of 2.25 g L^−1^ and 193 mg L^−1^ or volatile fatty acid concentrations of 0.75 g L^−1^were reliably identified which are lower than the commonly reported thresholds for critical process stages based on chemical parameters. Although a change in the microbiome structure does not necessarily indicate an upcoming critical process stage, the recorded community-level change points might be a first indication to carefully observe the process.

## 1. Introduction

Anaerobic digestion of livestock manure for the production of biogas provides a number of benefits creating interest in this technological application in many countries all over the world (e.g., [1,2,3,4]). Firstly, it produces methane (CH_4_) that can be used for the generation of electricity, heat and fuels and, as the production of biogas is independent from weather conditions, it can provide base-load power and holds the potential to balance fluctuating electricity supply from other renewable energy sources [5,6,7]. Secondly, it reduces greenhouse gas emissions from livestock husbandry (e.g., [8,9,10,11]) at relatively low mitigation costs [12,13,14] by substituting fossil fuels, avoiding CH_4_ and nitrous oxide (N_2_O) emissions from manure storage, replacing synthetic fertilizers and decreasing N_2_O emissions after field application of digestates [15,16]. Thirdly, anaerobic digestion of livestock manure improves organic fertilizer quality compared with undigested manure due to a better availability of important crop nutrients such as ammonium, phosphate and potassium and simultaneously improves soil structure and increases the soil organic matter content [17,18]. Moreover, biological degradation during anaerobic digestion can decrease the concentrations of weed seeds [19], pathogens [20,21] and antibiotics [21,22].

In Germany, for example, approximately 1080 biogas plants use livestock manure with a mass-based share of manure of at least 80% [23], whereby half of them are so called manure-based small biogas plants with an installed capacity of ≤75 kW_el_ [23]. Despite the benefits of using livestock manure for biogas production and the promotion by the Renewable Energy Act since 2009, still only 26.6% of the produced liquid cattle manure, 4.6% of solid cattle manure, 15.9% of swine manure and 13.4% of chicken/poultry manure were converted by anaerobic digestion in 2017 [24]. Causes for the currently low exploitation of the manure potential for biogas production lie in a number of specific challenges: Firstly, feedstock characteristics of some types of livestock manure are critical for the process of anaerobic digestion, e.g., the high lignocellulose contents of solid cattle and horse manure or the high nitrogen content of solid chicken/poultry manure [25,26,27,28,29]. Secondly, CH_4_ yields mainly depend on the livestock/animal type the manure derived from [30,31,32] and are generally lower compared to crops, especially energy crops [33]. Last, but not least, the high water content and low energy density of liquid livestock manure make the transport highly inefficient and thus require biogas plants close to the livestock facilities which often results in small-scale biogas plants with high specific investment cost for electricity generation [34].

Regarding the feedstock characteristics, e.g., of chicken manure, and the related risk of a process disturbance due to an elevated release of ammonium nitrogen and free ammonia (NH_4_^+^/NH_3_) [35,36,37], the objective of many studies was and still is to derive recommendations for an optimized process operation and to provide benchmarks that indicate impending critical process conditions. Often reported threshold values for process inhibition range between 3 to 5 g L^−1^ for NH_4_^+^ and 80 to 400 mg L^−1^ for NH_3_ [35,36,38,39]. There is evidence that certain members of the biogas microbiome, e.g., members of the bacterial phyla Bacteroidetes and Cloacimonetes, respond sensitively against elevated NH_4_^+^/NH_3_ concentrations, which is indicated by a significant decrease in their relative abundance or even their disappearance prior to an upcoming process disturbance [38,39,40]. However, it has also been shown that the biogas microbiome is enabled to adapt to higher NH_4_^+^/NH_3_ concentrations [41,42]. Nevertheless, the effects on the biogas microbiome are still not completely understood and hence the handling of feedstocks such as chicken manure is still critical and requires further research. Moreover, a process instability or disturbance is most often not related to one single cause/factor but rather to various cause/factors [37]. In this regard, higher process temperatures, for example, lead to an enhanced conversion of biomass into biogas per time unit due to a higher microbial activity, which subsequently also lead to an accelerated release of organic acids and other potentially process-inhibiting metabolites. Hence, a better understanding of the digestion process, particularly the underlying microbial diversity and how biogas microbiomes respond to management measures of the plant operators, can help to cope with unfavorable feedstock characteristics and to increase methane yields from livestock manure.

In order to elucidate the microbial diversity, various methods are available, ranging from isolation, cultivation and characterization of especially yet unknown microorganisms [43,44,45], through the detection of highly complex and dynamic microbial communities [39,46,47,48] right up to the application of multivariate statistics [49,50,51] and microbial network analyses [52,53,54]. Among the available methods, 16S rRNA gene amplicon sequencing is mostly used to compare different anaerobic digesters at a certain time while highlighting that the microbiome structure is significantly affected by the supplied feedstocks (available macro- and micronutrients), the process temperature as well as the NH_4_^+^/NH_3_ concentration [55,56,57,58]. However, long-term studies are highly important as they provide information about the microbiome response to changing environmental conditions [59,60], e.g., the management measures of anaerobic digestion plant operators. In this regard, a few studies showed that changes are not necessarily reflected by changes in the relative abundances of members of the so-called core microbiome, but rather in the variability of rarely occurring taxa, the transient microbiome [39,61,62,63]. Regarding this, established methods such as terminal restriction fragment length polymorphism (TRFLP) are still of great value, especially considering how microbial communities might respond to process instabilities and/or disturbances [38,39,46,64].

To the best of our knowledge, no studies are available so far which investigated the microbiome diversity of manure-based (small) biogas plants. Therefore, the objective of this study was to investigate the taxonomic microbiome structure of three manure-based small biogas plants and to relate their dynamic variations over time to the process performance. As the analyzed biogas plants differed in their digester types, their supplied feedstocks and their general process performance, we hypothesized (i) that the three investigated microbiomes varied in their structural composition according to the prevalent environmental conditions and (ii) that a comparison of the biogas plant specific microbiomes possibly allows the derivation of system-specific indicative taxa or groups of taxa for certain process conditions, e.g., due to the supplied feedstocks and their process-related parameters.

## 2. Materials and Methods

### 2.1. Characteristics of the Biogas Plants and Sampling of Digester Content

For this study, three manure-based small biogas plants (BPs) were analyzed monthly over a time period of one year between 2017 and 2018. The analyzed BPs differed in their reactor types/configuration, their supplied feedstocks, and their general process management (e.g., process temperature) (Table 1). Information on the general biogas plant characteristics as well as the amount of produced biogas were provided by the plant operators, while the organic loading rate (OLR) given as the influent volatile solids (VS) mass rate [kg_VS_] per unit volume [m^3^] and day [d] and the hydraulic retention time (HRT) given in days [d] were calculated based on the provided data (Table 1).

**Figure 1:**
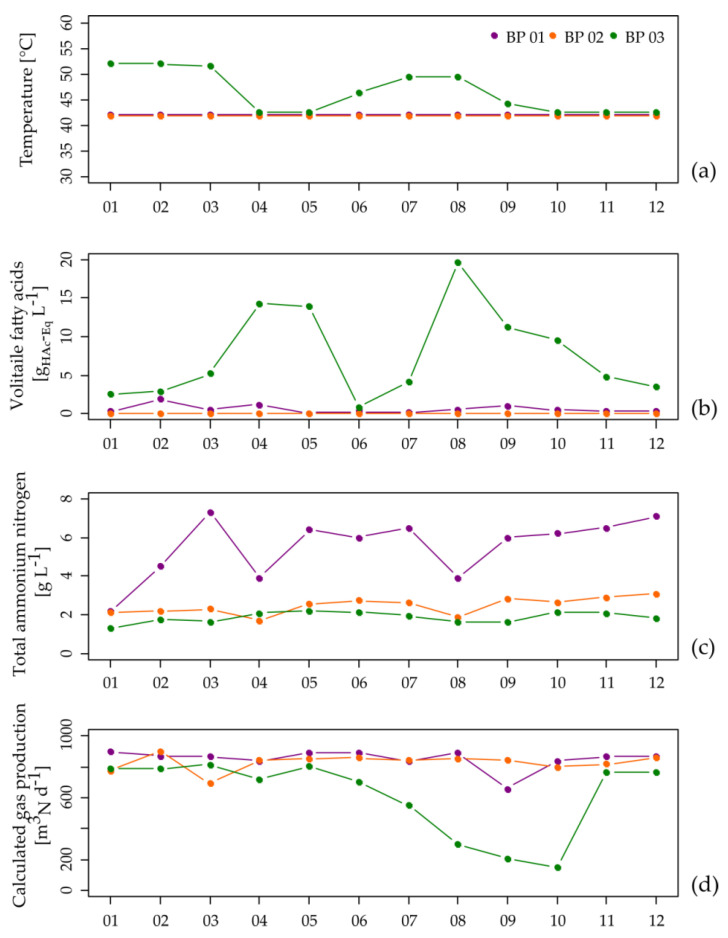
Process temperature (**a**), concentration of total volatile fatty acids (**b**) and total ammonium nitrogen (**c**) at the points of sampling of the analyzed main anaerobic digesters as well as the calculated biogas amounts of the entire biogas plants (**d**) over a time period of one year.

The two identically constructed biogas plants BP 01 and BP 02 consist of one heated (approximately 42 °C) digester with a volume of 847 m³ in series with a secondary unheated digester with a volume of 1742 m³ (all continuously stirred tank reactors, CSTRs). Overall, 654 and 450 m³ biogas can be stored in the gas-tight roof of each digester for BP 01 and BP 02, respectively. Beside these technological similarities, BP 01 and BP 02 differed in their feedstock composition: In addition to the main feedstocks liquid and solid cattle manure as well as maize and grass silage, BP 01 was fed with chicken manure (approximately 10% of the total feedstock amount), while in BP 02, horse manure was supplied (approximately 28% of the total feedstock amount) (Table 1 and Figure S1). The mean OLRs for BP 01 and BP 02 were 2.0 ± 0.3 and 3.2 ± 0.5 kg_VS_ m^−3^ d^−1^ with HRTs of 68.5 ± 5.3 and 50.2 ± 3.3 days, respectively (Figure S2). Compared to these two BPs, biogas plant BP 03 consists of a heated plug flow fermenter with a volume of 120 m^3^, followed by an unheated secondary digester (CSTR) with a volume of 1680 m³ which is insulated to maintain a temperature of approximately 30 °C. At all biogas plants, a combined heat and power unit produces 75 kW electricity and heat, whereby the electricity is fed into the grid and the heat is used for heating the fermenter and private houses. The main digester of BP 03 was operated at a mean temperature of 46.5 ± 4.1 °C (Table 1, Figure 1a) and fed with liquid and solid cattle manure (ca. 80% of the total feedstock amount without recirculation) and small amounts of maize grain flour silage as well as cattle feed residues consisting of maize and grass silage and minerals (Table 1 and Figure S1). Compared to BP 01 and BP 02, the OLR of BP 03 was 17.2 ± 2.1 kg_VS_ m^−3^ d^−1^, thus much higher, with a consequently much lower HRT of 7.2 ± 1.0 days (Figure S2). Due to these general process engineering variations, differences in the overall process performance, especially in the chemical process parameters, were expected.

Samples of the main digester content were taken monthly over one year according to Theuerl et al. [65], considering that the samples were characteristic for the digester content at the respective operational conditions. Samples were taken via a sampling nozzle/port, whereby the pipe section was purged at least twice. After sample taking, bottles were stored on ice to reduce the microbial activity, and directly transferred to the laboratory, where aliquots were taken for chemical and molecular biological analyses and immediately frozen at −20 °C until further analyses.

### 2.2. Chemical Analyses

The following chemical analyses were conducted on all taken samples: total solids (TS), volatile solids (VS), total ammonium nitrogen (TAN), various volatile fatty acids (VFA) and pH values according to standard methods [66]. The free ammonia (NH_3_) concentration was calculated as a function of the TAN concentration, the pH and the temperature, according to Hansen et al. [67]. In order to provide information on the feedstock use efficiency, the VS degradation degree was determined via mass balance calculations of the in- and output as the mean of the 12-month trial period in combination with residual methane potential tests of the digestate with a duration of 60 days at 37 °C according to VDI 4630 [68].

### 2.3. Inventory of the Taxonomic Microbiome Diversity

Samples which were used for the taxonomic microbiome inventory were taken after a three-month lasting lead time under stable process conditions.

In order to record the taxonomic microbiome diversity, in a first step, high-quality DNA was extracted in triplicates using the FastDNA^®^ spin kit for soil (MP Biomedicals, France) in combination with the Genomic DNA Clean & Concentrator^TM^ kit (Zymo Research, USA). Afterwards, 16S rRNA gene amplicon libraries were constructed by using the “16S Metagenomic Sequencing Library Preparation” protocol (Illumina Inc., San Diego, CA, USA). The universal primer pair Pro341F/Pro805R [69] was used to amplify the V3–V4 hypervariable region of the 16S rRNA genes with an average size of 460 base pairs (bp). The obtained amplicons were purified with magnetic beads (Agencourt AMPureXP, Beckman Coulter Genomics Inc., Brea, CA, USA), while adapter and index sequences were subsequently added (Nextera XT Index Kit, Illumina Inc., San Diego, CA, USA). The generated amplicon libraries were sequenced on the Illumina MiSeq platform applying the paired-end protocol for 300 bp reads. The bioinformatic pre-processing was performed by using a pipeline which includes a previous quality control of the raw sequencing reads with “FastQC” [70], the merging of the forward and reverse reads with “FLASH” [71] and the primer removal with “cutadapt” [72]. The program “sickle” [73] was used for quality trimming based on a quality value of 20 (Q20) and a base call accuracy of 99%. The resulting high-quality reads were subsampled to a given depth of 50,000 reads for every sample applying the tool “seqtk” (https://github.com/lh3/seqtk). All following processing steps were carried out within the QIIME platform which contains a broad range of different tools for high-throughput community sequencing data [74,75,76]. For an abundance- and reference-based detection of chimeric sequences, the program “usearch61” was used [77]. An open reference-based clustering of operational taxonomic unit (OTU) and a taxonomic assignment was done while using the 16S rDNA SILVA reference database [release 132, Apr. 2018]. The finally resulting taxa abundances of the triplicates of each sample were used to calculate the median which was then normalized to 100%. The raw 16S rRNA gene amplicon sequences are deposited in the European Nucleotide Archive (ENA) under the project PRJEB38916.

### 2.4. Microbial Community Profiling Using TRFLP and System Ecological Data Evaluation

To profile the dynamic variation of the occurring microbiomes over time, one of the most commonly applied and reliable fingerprinting techniques, TRFLP, was used [46]. As TRFLP analysis is based on a restriction digest of fluorescently labelled PCR products, in a first step, high-quality DNA was extracted (see Section 2.3). TRFLP analysis, in general, was carried out according to the protocol published by Klang et al. [78]. After DNA extraction, the 16S rRNA genes of bacteria and archaea were amplified (three replicates per crude DNA extract) with the primer pairs 27F/926MRr (bacteria) and Ar109f/Ar912r (archaea), whereby the forward primers were fluorescently labelled with Cy5 at the 5′-end. The PCR products were purified with the Nucleospin Gel and PCR Clean-up kit (Macherey und Nagel, Düren, Germany) and subsequently digested with MspI and Hin6I (bacteria) or with AluI (archaea), while the obtained restriction fragments were separated according to their lengths using the GenomeLab™ GeXP Genetic Analysis System (AB SCIEX Germany GmbH, Darmstadt, Germany). The obtained raw data were pre-analyzed with the GeXP analysis software (version 10.2), whereby only profiles (electropherograms) with an internal standard deviation of 0.39 nucleotides (nt) or less were considered for further analyses [79]. A detailed bioinformatic processing was then performed using the software package BioNumerics version 7.6 (Applied Maths, Kortrijk, Belgium) according to Klang et al. [78].

For the statistical analyses, only terminal restriction fragments (TRFs) with a relative abundance over 1% were used. All statistical analyses were carried out with the R Project for Statistical Computing [80] using the “vegan” package [81]. In order to evaluate the ecological diversity, meaning to validate the relationship between (groups of) biological entities (e.g., TRFs) and their environment (physical and/or chemical conditions), exploratory unconstrained analysis nonmetric multidimensional scaling (NMDS) was carried out [49,50,82]. The distance matrix for NMDS was calculated using the Bray–Curtis algorithm [83]. Environmental vectors were calculated using “envfit”, while the results were sorted according to the highest R^2^ values with *p* values of 0.001.

To define ecological thresholds where ecosystem attributes suddenly change due to small variations in specific environmental drivers, Threshold Indicator Taxa Analysis (TITAN) is a promising method as it provides not only the detection of potential indicator taxa, but rather as it provides information for potential community-level change points [51,84], whereby both (micro-) biological parameters such as taxa abundances [85,86] and chemical parameters such as targeted nutrient concentrations can be used [87]. TITAN was carried out using the R package “TITAN2” [51,84].

## 3. Results and Discussion

### 3.1. Performance of the Analyzed Manure-Based Small Biogas Plants

In general, BP 01 and BP 02 showed an overall stable process performance with low VFA concentrations (Figure 1b and Table S1), although BP 01 showed with 5.5 ± 1.5 g L^−1^ high TAN concentrations (Figure 1c and Table S1), corresponding to 1245 ± 530 mg L^−1^ NH_3_ which is caused by the feeding of chicken manure. Considering that values between 3 to 5 g L^−1^ (TAN) and 80 to 400 mg L^−1^ (NH_3_) are often reported as thresholds for process inhibition [35,36,38,39,40], the members of the microbiome of BP 01 seem to be well adapted to the elevated TAN/NH_3_ concentrations as they produce similar amounts of biogas as the microbiome of BP 02 (Figure 1d and Table S1). The extent of VS degradation for BP 01 and BP 02 was 53.0% and 63.4% in the main digester as well as 63.6% and 69.1% in the entire digestion system. The relative residual CH_4_ potentials, sampled at the secondary digesters, were 6.4% for BP 01 and 11.5% for BP 02. The higher relative residual CH_4_ potential for BP 02 is particularly caused by the shorter HRTs of this biogas plant (BP 01: 209 days, BP 02 153 days). However, the VS degradation degrees and the relative residual CH_4_ potentials for both biogas plants are in the expected range.

In contrast, BP 03 was characterized by a highly unstable process performance with VFA concentrations up to 19.6 g L^−1^ (Figure 1b, Table S1, and Figure S3). BP 03 was operated under thermophilic conditions (52 °C). Higher process temperatures lead to an increased microbial activity and hence more biomass is converted into biogas per time unit [40,88,89,90]. However, an increased metabolic activity is also associated with higher degradation rates resulting in an accelerated release of organic acids and other potentially process-inhibiting metabolites, which increases the risk of unstable process conditions [37]. Exactly these relationships were found in BP 03. As a response to the recorded increase in the VFA concentration of approximately 14 g L^−1^ (Figure 1b, Table S1, and Figure S3), the plant operator reduced the process temperature from 52 °C to 43 °C (Figure 1a) and simultaneously increased the amount of recirculate from the digestate storage tank from 10 to 15 m^3^ d^−1^ (Figure S1). Both modifications apparently led to process stabilization indicated by a decreasing VFA concentration over a period of two months (Figure 1b and Table S1). This might be related to the reduction in the overall microbial metabolic activity as well as the increased degradation efficiency, or in this case, most preferentially the degradation of organic acids by “starved” microorganisms [91,92] which were in this case already adapted to the mesophilic temperature (32.9 °C ± 4.1 °C within the digestate storage tank). Due to the supposed process stabilization, the plant operator increased the process temperature again (Figure 1a and Table S1), which directly resulted in a serious process disturbance indicated by a massive drop in the amount of produced biogas (Figure 1d and Table S1). The unfavorable process conditions were also reflected by the extent of VS degradation, especially in the main digester which was calculated to be 44.0%. Moreover, the relative residual CH_4_ potential was 24.9% in month nine of the 12-month monitoring period. A repeated test one month after the monitoring period showed the recovery of process stability and performance with a relative residual CH_4_ potential of 11.1%. This example shows that changes in the temperature regime should be performed with caution and slowly so that the microbial community can adapt to the new environmental conditions, particularly in the temperature range between 41 °C and 50 °C (e.g., [40,88]). The process disturbance most probably happened because the microbiome did not have enough time to fully regenerate (see Section 3.3). Only a further decrease in the process temperature to 43°C coupled with an increased amount of recirculate led to an appropriate stabilization of the process over a period of four months. In terms of process stability, the comparison of the three small-scale manure-based BPs shows that plant operation under thermophilic conditions is not necessarily favorable, particularly as higher decomposition rates may result in an enrichment of organic acids, while a stabilization might require the recirculation of relatively large amounts of digester contents from the post digesters into the main digester of the corresponding biogas plant.

To draw up an interim balance, the here presented study does not only provide information related to differences in biogas plant operation such as varying OLRs/HRTs or especially to the handling of potential process-critical feedstocks such as chicken manure, but it also enables the evaluation of a supposed long-lasting unstable process phase. Hence, two aspects can be addressed: (i) by comparing two well-running systems which differ in their general biogas plant operation, especially in their TAN/NH_3_ concentrations (caused by the use of chicken manure), this study enables to answer the question whether there is a point of change where biogas microbiomes potentially differentiate from each other and (ii) by comparing well-running systems with an unstable system, this study might provide information on microbiome representatives which are specific either for well-running systems or for disturbed systems including the potential to define a respective system change point.

### 3.2. Inventory of the Taxonomic Microbiome Diversity

**Figure 2:**
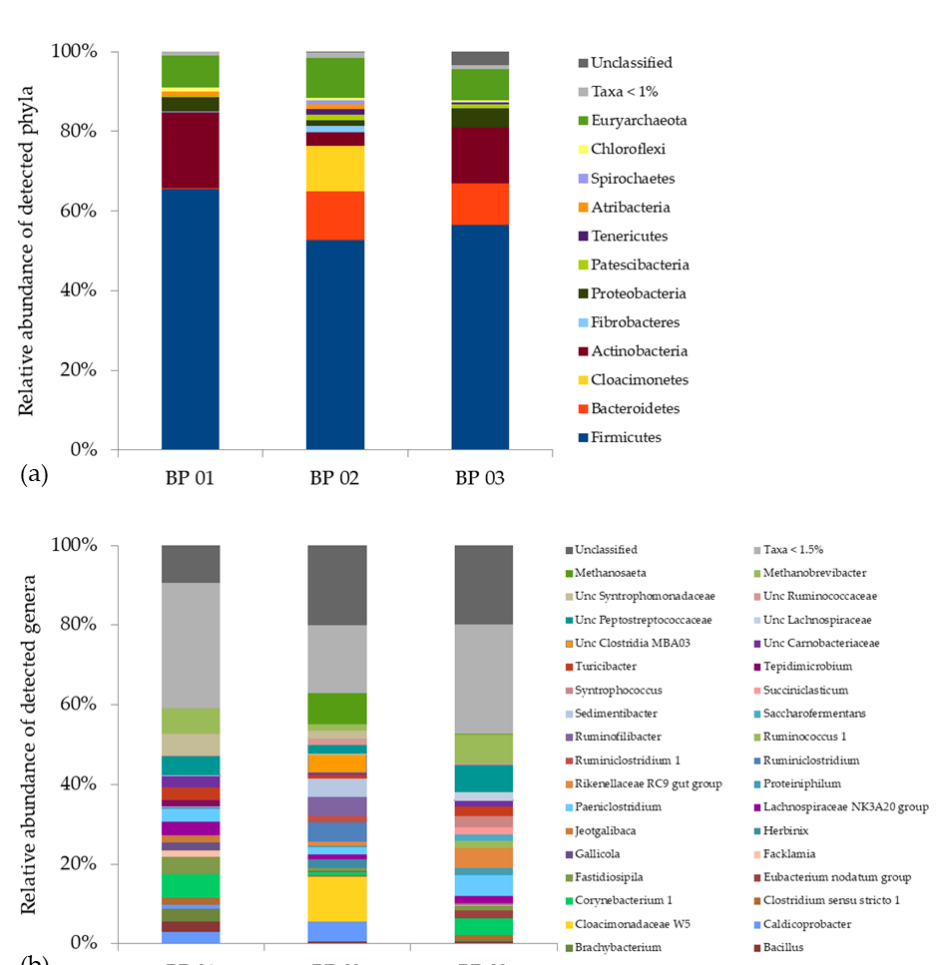
Taxonomic profiles of the main fermenter microbiomes of the analyzed biogas plants BP 01, BP 02 and BP 03 at the phylum (**a**) and genus (**b**) level after a three-month lasting lead time.

A total of 2297 OTUs were identified in the analyzed anaerobic digesters, whereas BP 01 contained 1451 OTUs, BP 02 1248 OTUs and BP 03 1510 OTUs. The taxonomic profiles showed an average of 90.2% ± 1.6% from the domain Bacteria, 8.6% ± 1.4 % from the domain Archaea and 1.2% ± 1.9% of microorganisms that have not yet been classified. The most abundant phyla were Firmicutes (58.2% ± 6.7%), Actinobacteria (12.2 ± 8.1%), Bacteroidetes (7.6% ± 6.6%), Cloacimonetes (3.8% ± 6.6%), Proteobacteria (3.3% ± 1.6%) and Euryarchaeota (8.6% ± 1.3%), which generally is in accordance with previously published studies [55,57,61,93]. However, an in-depth look into the taxonomic profiles revealed distinct variation in the microbiome structure within the analyzed BPs (Figure 2a,b and Table S2).

In BP 01, 92.0% of the recorded OTUs were assigned to the domain Bacteria and 7.9% to the domain Archaea. The most abundant phyla were Firmicutes (65.6%), Actinobacteria (19.1%), Proteobacteria (3.7%), Atribacteria (1.1%), Chloroflexi (1.0%) and Euryarchaeota (7.9%) (Figure 2a) with the predominant genera *Brachybacterium* (3.1%), *Corynebacterium* (5.6%), *Fastidiosipila* (4.4%), “*Lachnospiraceae* NK3A20” (3.2%), *Paeniclostridium* (3.3%), *Turicibacter* (3.3%), “*uncultured Peptostreptococcaceae*” (4.8%), “*uncultured Syntrophomonadaceae*” (5.4%) and *Methanobrevibacter* (6.3%) (Figure 2b). Most notable is the presence of representatives from the phylum Proteobacteria (3.7%) and in particular the phylum Atribacteria (1.1%). So far, there is no pure or enriched culture described for a member of the phylum Atribacter. However, metagenome and single-cell-genome studies showed that members of this phylum are supposed to occur in habitats that contain considerable amounts of organic carbon but have a relatively low availability of inorganic compounds [94] which are essential for living under strictly anaerobic conditions. Hence, the assumed fermentative and/or syntrophic metabolic strategies of members of the phylum Atribacteria such as the genus “*Candidatus Caldatribacterium*” are crucial for survival [94]. In addition, the relatively high share of representatives of the phylum Proteobacteria indicates that syntrophic lifestyles might play an important role in this anaerobic digestion system, as this phylum includes genera such as *Syntrophobacter*, *Syntrophus* and *Smithella*, which are known to convert C3–C6 fatty acids (e.g., propionic acid, butyric acid) into carbon dioxide (CO_2_) and hydrogen (H_2_), which are subsequently converted into methane by hydrogenotrophic archaea [37,95,96]. This coincides with the high relative abundance of members of the archaeal genus *Methanobrevibacter* (6.3%) recorded for this BP (Figure 2b). Taking into account that BP 01 was fed with chicken manure resulting in high TAN/NH3 concentrations (see Section 3.1), the occurrence of (obligate) acetoclastic methanogens was impossible or restricted as they are highly sensitive to elevated TAN/NH_3_ concentrations [36,97,98].

In BP 02, 89.7% of the recorded OTUs were assigned to the domain Bacteria and 10.2% to the domain Archaea. The most abundant phyla within the microbiome were Firmicutes (52.6%), Bacteroidetes (12.3%), Cloacimonetes (11.5%), Actinobacteria (3.4%) and Euryarchaeota (10.1%) (Figure 2a). Compared to BP 01, most notable for this BP are the high relative abundances of members of the both phyla Bacteroidetes and Cloacimonetes, which are assumed to be microbial indicators for a well-running mesophilic system, if they occur together with members from the archaeal genus *Methanosaeta* (syn. *Methanotrhix*) [65], which were found with a relative abundance of 7.8%. The question arises whether members of these groups are sensitive to unfavourable process conditions, e.g., elevated TAN/NH_3_ concentrations caused by the supply of supposedly high amounts of grass silage and chicken manure (comparing BG 01 with BP 02), and hence whether they are potential indicative taxa which can be used to elucidate points where ecosystem traits/features suddenly change (see Section 3.3).

In BP 03, 89.0% of the recorded OTUs were assigned to the domain Bacteria and 7.7% to the domain Archaea. The most abundant phyla within the microbiome were Firmicutes (56.5%), Actinobacteria (14.2%), Bacteroidetes (10.4%), Proteobacteria (4.7%) and Euryarchaeota (7.7%) (Figure 2a) with the predominant genera *Corynebacterium* (4.7%), *Paeniclostridium* (5.1%), “*Rikenellaceae RC9 gut group*” (4.9%), *Syntrophococcus* (2.7%), *Turicibacter* (2:5%), “*uncultured Lachnospiraceae*” (2.1%), “*uncultured Peptostreptococcaceae*” (6.7%) and *Methanobrevibacter* (7.2%) (Figure 2b).

Besides the mentioned differences, common to all three biogas plants is the occurrence of representatives from the phyla Firmicutes and Actinobacteria. While members of the phylum Firmicutes are frequently found within anaerobic digesters, members of the phylum Actinobacteria are far less recognized. Representatives of this phylum are well-known for two reasons: (i) as important pathogens causing serious diseases such as tuberculosis, leprosy or diphtheria and (ii) as important producers of drugs, particularly antibiotics, considerably reducing the impact of infectious diseases in the last century [99]. However, during recent years, their relevance as decomposers of supposed recalcitrant organic matter, especially lignocellulose, and hence their potential to contribute to the production of bio-based products (energy and materials) while reducing carbon emission have come into consideration [99,100]. Hence, their high relative abundance within the microbiomes of the analyzed biogas plants, especially within BG 01 with almost 20%, is probably related to their role in hydrolyzing complex carbohydrates and/or nitrogenous compounds derived from the grass silage and chicken manure. There are indications that members of the phylum Actinobacteria might specifically occur in anaerobic digestion systems with higher amounts of excrements (manure from livestock husbandry or wastewater sludge) as revealed by a comparison with previously published studies [55,61,93,101]. However, a comprehensive (meta-) study would be necessary considering the microbiome structure of various digestion system as well as of the gastrointestinal tract of various animals in order to significantly elucidate their system specificity and the origin they derive from.

### 3.3. Dynamic Variation of the Microbial Community Over Time

**Figure 3:**
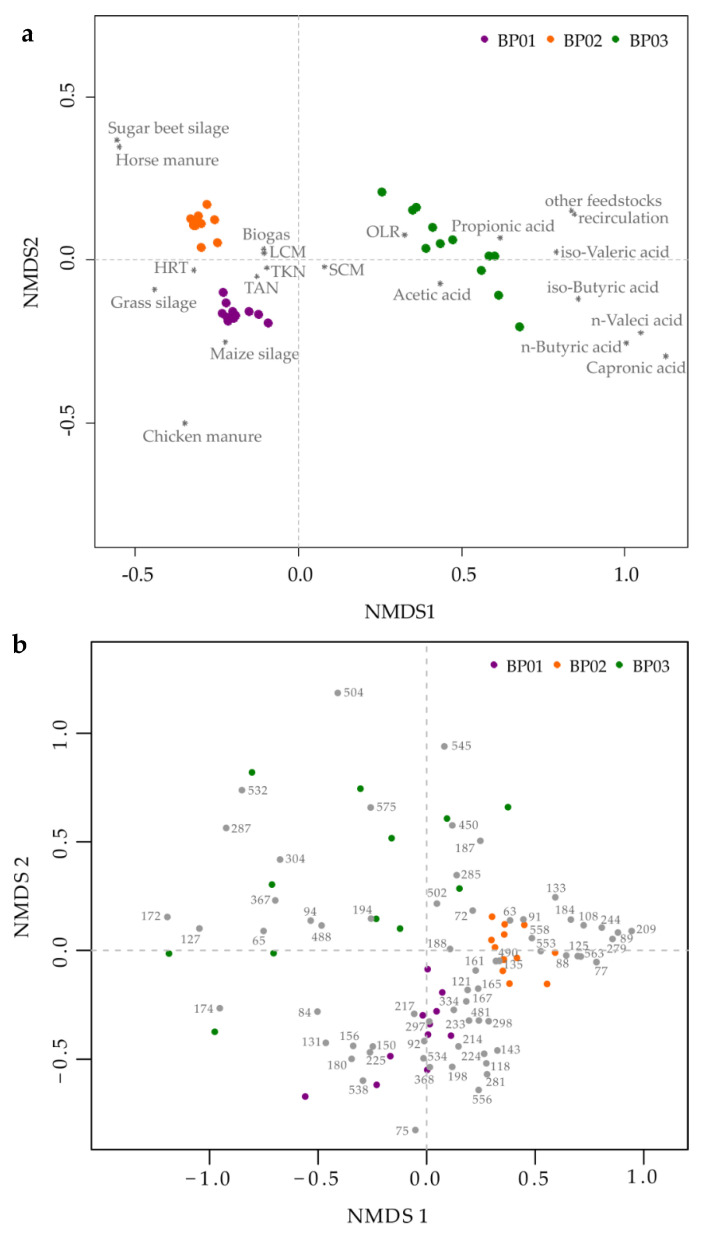
Non-metric multidimensional scaling (NMDS) analysis based either on the prevalent process engineering and process chemical parameters (**a**) or the detected bacterial terminal restriction fragments (given in gray dots with numbers) (**b**) of the three analyzed manure-based small biogas plants. Each colored dot symbolizes one sampling point of the respective biogas plant. Marked with a star (∗) are abiotic parameters with *p* = 0.001. OLR = organic loading rate, HRT = hydraulic retention time, TAN = total ammonium nitrogen, TKN = total Kjeldahl nitrogen, LCM = liquid cattle manure, SCM = solid cattle manure.

In order to elucidate the dynamic variations of the microbial communities over time, especially for BP 03, TRFLP analyses were carried out separately for the bacterial and archaeal communities. The temporal microbiome profiles were related to the prevalent abiotic parameters (e.g., feedstock supply, OLR, HRT, process temperature, VFA, TAN/NH_3_, produced biogas amount) by NMDS analyses [49,50,82] as shown in Figure 3.

As expected, the NMDS analysis based on the process engineering and process chemical parameters revealed a clear separation of the three small-scale manure-based BPs (Figure 3a). As indicated by the compressed sampling point location of BP 01 and BP 02 within the ordination plot, the underlying datasets of each BP were quite similar, suggesting a stable process performance over time. However, BP 01 and BP 02 were significantly affected by the feedstocks chicken manure and horse manure, resulting in a separate clustering of both BPs. These two feedstocks seem to have a significant impact on the structural composition of the microbial community, particularly at the archaeal level (Figure S4). During the entire sampling period, BP 01 had a more diverse archaeal community including members of the genera *Methanobacterium*/*Methanobrevibacter* (TRF342), *Methanomassiliicoccus* (TRF470) and *Methanosarcina* (TRF625), while BP 02 showed a clear dominance (≥ 60 %) of the genus *Methanosaeta* (TRF106) (Figure S4). Considering that members of the genus *Methanosaeta* dominate anaerobic digestion systems at low concentrations of VFA and TAN (100-150 mg L^−1^ and 3.0 g L^−1^, respectively) [98], the high relative abundance in BP 02 was not surprising as both the VFA and the TAN concentrations were with 64 ± 35 mg L^−1^ and 2.5 ± 0.4 g L^−1^ under the threshold values. In addition, Theuerl et al. [65] investigated the microbiome structures of 36 anaerobic digestion microbiomes originating from 22 BPs and assumed that the occurrence of members from the archaeal genus *Methanosaeta* (syn. *Methanothrix*) are indicators for stable process conditions if they are co-detected with members from the bacterial phyla Bacteriodetes and Cloacimonetes. For BP 02, the NMDS analysis, for example, revealed that TRF89 and TRF91 (both phylum Bacteroidetes, order Bacteroidales) as well as TRF161 (phylum Cloacimonetes) were significantly related (R^2^ > 0.6, *p* = 0.001) to the respective microbiome structure (Figure 3b).

In comparison with BP 01 and BP 02, very unstable process conditions over time were recorded for BP 03 (see Section 3.1), which are reflected in a high degree of variability within the microbiome structure over time (Figure 3b and Figure S4). As already mentioned, BP 03 was actually planned to be operated under thermophilic conditions (52 °C) which is generally related to a higher biomass degradation rate due to a higher metabolic activity of the occurring microorganisms but also to an accelerated release of organic acids or other process-inhibiting metabolites [37]. Due to an increase in the VFA concentration (Figure 1b) which was accompanied by changes in the structural composition of the microbiome (Figure S4), the plant operator reduced the process temperature to 42°C and increased the amount of recirculate from the digestate storage tank. Both modifications apparently led to process stabilization, indicated by a decreased VFA concentration (Figure 1b), whereby the microbial community seemed to be resilient [102] and was thus assumed to return to its original state after the process instability (Figure S4). Considering the microbiome structure at the time of the supposed stable process phase (time points 06/07 in Figure S4), it is noticeable, particularly at the bacterial level, that the microbiome had not yet fully stabilized again (comparison with time points 01–03 in Figure S4). Hence, the following process disturbance due to a temperature increase was retrospectively not surprising. These findings are in accordance with other studies, which examined how changes in the temperature regime affect the structure of the microbial community and the associated process stability, revealing that unstable process conditions have to be expected, particularly in the temperature range between 44 °C and 50 °C [40,88]. The risk of a process disturbance was enhanced by the plant operator because he did not give the microbial community enough time to regenerate/stabilize after the first instability. Only a further temperature decrease, coupled with an increase in the amount of recirculate, led to an appropriate stabilization of the microbiome structure and hence of the process performance over a period of four months, whereby the structural composition of the microbial community still remained highly variable until the end of the monitoring period (Figure S4).

However, in order to ensure a high process stability and efficiency with low susceptibility to disturbances [37], the challenge is to define the point at which a certain system attribute, for example, the VFA or TAN/NH_3_ concentration, switches corresponding to a distinct change in the microbiome structure or in particular in indicative microbiome representatives.

### 3.4. Community-Level Change Points and Potential Indicator Taxa

**Figure 4:**
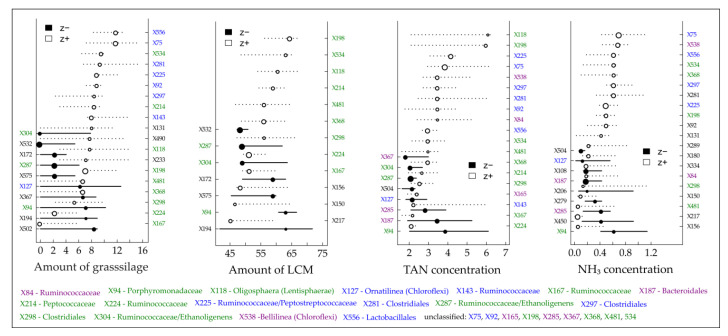
Threshold Indicator Taxa Analysis (TITAN) of the individual density of detected terminal restriction fragments (TRFs) in response to the gradient of the amount of grass silage and liquid cattle manure (LCM) as well as the concentrations of total ammonium nitrogen (TAN) and ammonia nitrogen (NH_3_). Circles represent the change points of indicative TRFs that decreased (black, negative response, left y-axis) or increased (white, positive response, right y-axis) with increasing gradients and are sized based on the magnitude of the response (z-score; the larger the circle, the stronger the response). Horizontal lines represent 5th and 95th quantiles of 500 bootstrap replicates. Color code: green = TRFs recorded in all four gradients, blue = TRFs recorded in the gradients for the amount of grass silages as well as the concentration of TAN and NH_3_, violet = TRFs recorded for the gradients of the TAN and NH_3_concentrations.

**Figure 5:**
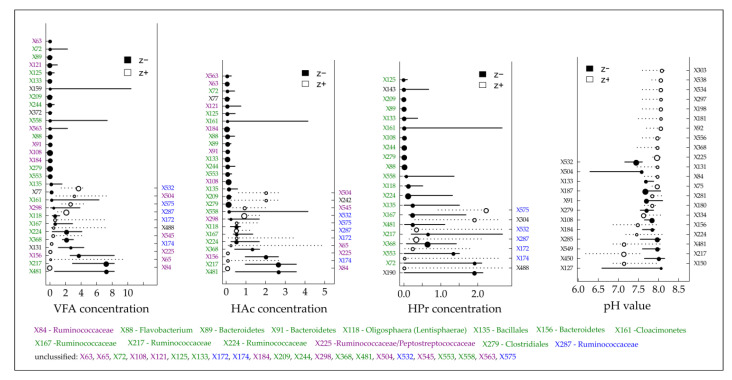
Threshold Indicator Taxa Analysis (TITAN) on the individual density of detected terminal restriction fragments (TRFs) in response to the gradients of the total volatile fatty acids (VFA), acetic acid (HAc), propionic acid (HPr) concentration and the pH value. Circles represent the change points of indicative TRFs that decreased (black, negative response, left y-axis) or increased (white, positive response, right y-axis) with increasing gradients and are sized based on the magnitude of the response (z-score; the larger the circle, the stronger the response). Horizontal lines represent 5th and 95th quantiles of 500 bootstrap replicates. Color code: green = negative responding TRFs in all gradients except pH value, blue = positive responding TRFs in all gradient except pH value, violet = negative/positive responding TRFs for VFA and HAc concentration.

One main objective was to derive community-level and indicative taxa change points for certain process parameters and hence to provide benchmarks that indicate impending critical process conditions. By using TITAN, change points for several bacterial TRFs responding negatively (decreasing abundances) or positively (increasing abundances) to elevated TAN/NH_3_ and VFA concentrations were determined (Figure 4 and Figure 5, Table S3).

TITAN revealed the occurrence of reliable indicative TRFs which are either negatively or positively correlated with increasing amounts of grass silage and/or liquid cattle manure and in particular with increasing TAN and NH_3_ concentrations (Figure 4). A certain number of indicative TRFs could be identified for all tested “environmental” gradients (marked in green in Figure 4). Considering the threshold values for process inhibition of 3–5 g L^−1^ for TAN and 80–400 mg L^−1^ for NH_3_ [35,36,38,39], previous and current research aimed at defining recommendations for an optimized plant operation by providing values which indicate upcoming critical process states. In this regard, TITAN recorded with 2.20 (negative response) and 3.00 g L^−1^ (positive response), intersecting at 2.25 g L^−1^ for the TAN concentration and with 331 (negative response) and 625 mg L^−1^ (positive response), intersecting at 193 mg L^−1^ for the NH_3_ concentration (Table S3) community-level change points below the commonly reported process inhibition thresholds. Of course, a change in the microbial community structure does not necessarily indicate an upcoming critical process stage as the microbiomes can adapt to elevated TAN and NH_3_ concentrations with an overall stable biogas production [39,41,42], which was also found for BP 01. However, the recorded community-level change points might be a first indication to be attentive.

Considering an overload of the microbial degradation potential which is often accompanied by an acid accumulation [37], TITAN revealed that the majority of reliable indicative taxa (symbolized by TRFs on the left y-axis in Figure 5) responded negatively to an increasing total VFA concentration and particularly to increasing concentrations of acetic and propionic acid. Most of them responded highly sensitive as indicated by the low change point values (Figure 5). Among the highly sensitive responding TRFs, almost all assignable TRFs belong to the two phyla Bacteroidetes (TRF X88, X89, X91 and X156) and Cloacimonetes (TRF X161) which again supports the assumption of Theuerl et al. [65] that these taxa might be indicators of well-running mesophilic anaerobic digestion systems. However, community-level change points were recorded at 0.15 (negative response) and 1.55 g L^−1^ (positive response) for the VFA concentration, at 0.12 (negative response) and 1.16 g L^−1^ (positive response) for the acetic acid concentration and at 0.04 (negative response) and 1.94 g L^−1^ (positive response) for the propionic acid concentration (Table S3). Both curves intersect at 0.75 g L^−1^ (VFA), 0.56 g L^−1^ (acetic acid) and 0.3 g L^−1^ (propionic acid), the points after which the sharpest changes within the microbiome structure occur. Based on the presented results, it can be assumed that well-running systems are characterized by organic acid values near the detection limit (as it was recorded for BP 02), whereby values higher than 0.75 g L^−1^ for total VFA concentration might be indicative of being aware of an upcoming critical process stage due to the sharp change within the microbiome structure. Although this is in contrast to Drosg [103], who postulated that VFA values only higher than 4.0 g L^−1^ indicate unfavorable process conditions, the presented results are not surprising as most chemical parameters which indicate process instabilities/disturbances (VFA, TAN/NH_3_, H_2_S or H_2_) result from previous microbial activities [37]. Consequently, changes in the microbiome structure are detected earlier. However, the question arises which taxa out of the entire microbiome are indicative for an upcoming critical process stage. So far, there is no reliable evidence whether the positively or the negatively responding taxa which occur after the intersect point along the increasing gradient might be indicators as a TITAN analysis for the amount of produced biogas did not give valid results (Figure S5).

Summarizing, the question remains whether community-level change points and recorded positively or negatively responding taxa might be indicators for upcoming critical process stages. Promising might be an approach which considers all reliable indicative taxa which respond negatively to, e.g., increasing VFA or elevated TAN/NH_3_ concentrations after the point where the community-level change points for negative and positive responses intersect, as this indicates the point at which there is a distinct change in a particular ecosystem attribute. In contrast and with special emphasis on elevated TAN/NH_3_ concentrations, strongly positively responding reliable indicative taxa could be evidence for a well-adapted system with an overall stable production of sufficient amounts of biogas. However, further research is needed to verify the here presented results.

## 4. Conclusions

The microbiomes of the three manure-based small-scale biogas plants differed significantly depending on feedstocks and process conditions. However, common to all three biogas plants was the occurrence of the phylum Actinobacteria with high abundances of up to 19.1% which might be a specific characteristic of manure-fed biogas plants. Reliable values for ecological change points in terms of distinct change points for both the entire communities and specific microbiome taxa at TAN and NH_3_ concentrations of 2.25 g L^−1^ and 193 mg L^−1^ or at VFA concentrations of 0.75 g L^−1^ were identified. These values are lower than the commonly reported thresholds for critical process stages based on chemical parameters, which supports the assumption that microorganisms show an earlier response to changing process conditions since the chemical parameters result from previous microbial activities. Specific microbiome members, particularly of the phyla Bacteroidetes and Cloacimonetes responded highly sensitively to increasing TAN/NH_3_ or VFA concentrations and hence can potentially be used in order to assess the process performance. However, further research with broader data sets (e.g., a representative number of well-running and unstable digesters, longer monitoring phases, a deeper taxonomic and functional microbiome profiling) is needed to verify the results presented here.

## Figures and Tables

**Table 1:** Summary of process engineering parameters of the main anaerobic digesters of the analyzed manure-based small biogas plants (BPs). Given are the mean values and their standard deviation as well as in brackets the percentage of each feedstock on the total feedstock amount as median value over a period of one year (one sampling date per month, n = 12). CSTR = continuously stirred tank reactor, PF = plug flow fermenter, FM = fresh mass, OLR = organic loading rate, HRT = hydraulic retention time, VS = volatile solids. For details see Table S1.

BP (Fermenter Type)	Feedstock Supply [t_FM_ d^−1^]	OLR/HRT [kg_VS_ m^−3^ d^−1^]/[d]	Temperature [°C]
BP 01 (CSTR)	Maize silage: 0.6 ± 0.2 (5.1%) Grass silage: 1.6 ± 0.4 (14.6%) Liquid cattle manure: 8.3 ± 0.4 (65.8%) Solid cattle manure: 0.7 ± 0.5 (5.7%) Chicken manure: 1.1 ± 0.2 (9.7%)	OLR: 2.0 ± 0.4 HRT: 77.2 ± 5.8	41.9 ± 0.1
BP 02 (CSTR)	Maize silage: 0.1 ± 0.1 (0.5%) Grass silage: 1.3 ± 0.2 (8.2%) Sugar beet silage: 0.1 ± 0.1 (0.5%) Liquid cattle manure: 11.0 ± 0.4 (64.8%) Solid cattle manure: 0.3 ± 0.1 (1.6%) Horse manure: 4.2 ± 1.3 (27.8%)	OLR: 1.9 ± 0.5 HRT: 58.7 ± 5.6	41.9 ± 0.0
BP 03 (PF)	Liquid cattle manure: 13.5 ± 1.8 (49.5%) Solid cattle manure: 2.1 ± 0.6 (8.2%) Others ^1^: 1.3 ± 0.5 (3.8%) Recirculate: 13.2 ± 2.5 (56.6%)	OLR: 17.2 ± 2.1 HRT: 7.2 ± 1.0	46.5 ± 4.1

^1^ Maize grain flour silage, cow feed residues consisting of maize and grass silage and minerals.

## Supplementary Materials

The following are available online at https://www.mdpi.com/2076-2607/8/8/1169/s1, Figure S1: Feedstock composition of the analyzed manure-based small biogas plants BP 01 (a), BP 02 (b) and BP 03 (c) over a time period of one year, Figure S2: Organic loading rate (a) and the corresponding hydraulic retention time (b) of the analyzed manure-based small biogas plants over a time period of one year, Figure S3: Recorded volatile fatty acid spectrum of the analyzed manure-based small biogas plants BP 01 (a), BP 02 (b) and BP 03 (c) over a time period of one year. To be considered: different axis scaling, Figure S4: Distribution diagrams of the recorded bacterial (a, c, e) and archaeal (b, d, f) terminal restriction fragment (TRFs) based on their relative abundance within the three investigated manure-based small biogas plants BP 01 (a, b), BP 02 (c, d) and BP 03 (e, f), Figure S5: Threshold Indicator Taxa Analysis (TITAN) on the individual density of detected terminal restriction fragments (TRFs) in response to the amount of produced biogas. Circles represent the change points of indicative TRFs that decreased (black, negative response, left y-axis) or increased (white, positive response, right y-axis) with increasing “environmental” gradients and are sized based on the magnitude of the response [z-score; the larger the circle, the stronger the response]. Horizontal lines represent 5th and 95th quantiles of 500 bootstrap replicates, Table S1: Summary of process engineering and process chemical parameters of the main anaerobic digesters of the analyzed manure-based small biogas plants over the entire sampling period. FM = fresh mass, OLR = organic loading rate, HRT = hydraulic retention time, ns = not supplied, TS = total solids, VS = volatile solids, TAN = total ammonium nitrogen, NH_3_ = free ammonia nitrogen, HAc = acetic acid, HPr = propionic acid, HiB = iso-butyric acid, HnB, n-butyric acid, HiV = iso-valeric acid, HnV = n-valeric acid, HC = capronic acid, VFA = volatile fatty acids, CH_4_ = methane, Table S2: Taxonomic profiles at the phylum and genus level of the main anaerobic digesters of the analyzed manure-based small biogas plants after a three month lasting lead time, Table S3: Community-level change points (ComCP) for selected operational and chemical process parameters based on the performed Threshold Indicator Taxa Analysis (TITAN) for negative (fsum[z-]) and positive (fsum[z+]) responders using only those taxa (terminal restriction fragment, TRFs) that are determined to be pure and reliable indicators. VFA = volatile fatty acids, HAc = acetic acid, HPr = propionic acid, LCM = liquid cattle manure, TAN = total ammonium nitrogen, NH_3_ = ammonia nitrogen.

## References

[1] Ahammad S.Z., Sreekrishnan T.R., Soccol C.R., Brar S.K., Faulds C., Ramos L.P. (2016). Biogas: An Evolutionary Perspective in the Indian Context. Green Fuels Technology.

[2] Chen B., Hayat T., Alsaedi A. (2017). Biogas Systems in China.

[3] Kemausuor F., Adaramola M.S., Morken J. (2018). A review of commercial biogas systems and lessons for Africa. Energies.

[4] Tabatabaei M., Ghanavati H. (2018). Biogas—Fundamentals, Process, and Operation.

[5] Hahn H., Krautkremer B., Hartmann K., Wachendorf M. (2014). Review of concepts for a demand-driven biogas supply for flexible power generation. Renew. Sustain. Energy Rev..

[6] Lauer M., Thrän D. (2018). Flexible biogas in future energy systems - Sleeping beauty for a cheaper power generation. Energies.

[7] Theuerl S., Herrmann C., Heiermann M., Grundmann P., Landwehr N., Kreidenweis U., Prochnow A. (2019). The future agricultural biogas plant in Germany: A vision. Energies.

[8] Agostini A., Ferdinando Battini F., Giuntoli J., Tabaglio V., Padella M., Baxter D., Marelli L., Amaducci S. (2015). Environmentally sustainable biogas? The key role of manure co-digestion with energy crops. Energies.

[9] Dalgaard T., Olesen J.E., Petersen S.O., Petersen B.M., Jørgensen U., Kristensen T., Hutchings N.J., Gyldenkærne S., Hermansen J.E. (2011). Developments in greenhouse gas emissions and net energy use in Danish agriculture e How to achieve substantial CO2 reductions?. Environ. Pollut..

[10] Meyer-Aurich A., Schattauer A., Hellebrand H.-J., Klauss H., Plöchl M., Berg W. (2012). Impacts of uncertainties on greenhouse gas mitigation potential of biogas production from agricultural resources. Renew. Energy.

[11] Weiske A., Vabitsch A., Olesen J.E., Schelde K., Michel J., Friedrich R., Kaltschmitt M. (2006). Mitigation of greenhouse gas emissions in European conventional and organic dairy farming. Agric. Ecosyst. Environ..

[12] Agostini A., Battini F., Padella M., Giuntoli J., Baxter D., Marelli L., Amaducci S. (2016). Economics of GHG emissions mitigation via biogas production from sorghum, maize and dairy farm manure digestion in the Po valley. Biomass Bioenerg..

[13] Kalt G., Lauk C., Mayer A., Theurl M.C., Kaltenegger K., Winiwarter W., Erb K.-H., Matej S., Haberl H. (2020). Greenhouse gas implications of mobilizing agricultural biomass for energy: A reassessment of global potentials in 2050 under different food-system pathways. Environ. Res. Lett..

[14] Scholz L., Meyer-Aurich A., Kirschke D. (2011). Greenhouse gas mitigation potential and mitigation costs of biogas production in Brandenburg, Germany. AgBioForum.

[15] Kaparaju P., Rintala J. (2011). Mitigation of greenhouse gas emissions by adopting anaerobic digestion technology on dairy, sow and pig farms in Finland. Renew. Energy.

[16] Massé D.I., Talbot G., Gilbert Y. (2011). On farm biogas production: A method to reduce GHG emissions and develop more sustainable livestock operations. Anim. Feed Sci. Technol..

[17] Arthurson V. (2009). Closing the global energy and nutrient cycles through application of biogas residue to agricultural land–Potential benefits and drawbacks. Energies.

[18] Valentinuzzi F., Cavani L., Porfido C., Terzano R., Pii Y., Cesco S., Marzadori C., Mimmo T. (2020). The fertilising potential of manure-based biogas fermentation residues: Pelleted vs. liquid digestate. Heliyon.

[19] Baute K.A., Robinson D.E., van Eerd L.L., Edson M., Sikkema P.H., Gilroyed B.H. (2016). Survival of seeds from perennial biomass species during commercial-scale anaerobic digestion. Weed Res..

[20] Fröschle B., Heiermann M., Lebuhn M., Messelhäusser U., Plöchl M. (2015). Hygiene and sanitation in biogas plants. Adv. Biochem. Eng. Biotechnol..

[21] Insam H., Gomez-Brandon M., Ascher J. (2015). Manure-based biogas fermentation residues—Friend or foe of soil fertility?. Soil Biol. Biochem..

[22] Massé D.I., Saady N.M.C., Gilbert Y. (2014). Potential of biological processes to eliminate antibiotics in livestock manure: An overview. Animals.

[23] Daniel-Gromke J., Rensberg N., Denysenko V., Stinner W., Schmalfuß T., Scheftelowitz M., Nelles M., Liebetrau J. (2018). Current developments in production and utilization of biogas and biomethane in Germany. Chem. Ing. Tech..

[24] German Environment Agency [GEA]—UNFCCC-Submission (2019). Submission under the United Nations Framework Convention on Climate Change and the Kyoto Protocol 2019, National Inventory Report for the German Greenhouse Gas Inventory 1990–2017.

[25] Budde J., Prochnow A., Plöchl M., Suárez T., Heiermann M. (2016). Energy balance, greenhouse gas emissions, and profitability of thermobarical pretreatment of cattle waste in anaerobic digestion. Waste Manag..

[26] Fuchs W., Wang X., Gabauer W., Ortner M., Li Z. (2018). Tackling ammonia inhibition for efficient biogas production from chicken manure: Status and technical trends in Europe and China. Renew. Sustain. Energy Rev..

[27] Hadin Å. (2018). From waste problem to renewable energy resource – exploring horse manure as feedstock for anaerobic digestions. Doctor of Philosophy.

[28] Mönch-Tegeder M., Lemmer A., Oechsner H., Jungbluth T. (2013). Investigation of the methane potential of horse manure. Agric. Eng. Int.: CIGR J..

[29] Morozova I., Nikulina N., Oechsner H., Krümpel J., Lemmer A. (2020). Effects of increasing nitrogen content on process stability and reactor performance in anaerobic digestion. Energies.

[30] Budde J., Heiermann M., Suárez Quiñones T., Plöchl M. (2014). Effects of thermobarical pretreatment of cattle waste as feedstock for anaerobic digestion. Waste Manag..

[31] Cu T.T.T., Nguyen T.X., Triolo J.M., Pedersen L., Le V.D., Le P.D., Sommer S.G. (2015). Biogas production from vietnamese animal manure, plant residues and organic waste: Influence of biomass composition on methane yield. Asian-Australas. J. Anim. Sci..

[32] Kafle G.K., Chen L. (2016). Comparison on batch anaerobic digestion of five different livestock manures and prediction of biochemical methane potential (BMP) using different statistical models. Waste Manag..

[33] Herrmann C., Idler C., Heiermann M. (2016). Biogas crops grown in energy crop rotations: Linking chemical composition and methane production characteristics. Bioresour. Technol..

[34] Scheftelowitz M., Thrän D. (2016). Unlocking the energy potential of manure - An assessment of the biogas production potential at the farm level in Germany. Agriculture.

[35] Rajagopal R., Massé D.I., Singh G. (2013). A critical review on inhibition of anaerobic digestion process by excess ammonia. Bioresour. Technol..

[36] Westerholm M., Moestedt J., Schnürer A. (2016). Biogas production through syntrophic acetate oxidation and deliberate operating strategies for improved digester performance. Appl. Energy.

[37] Theuerl S., Klang J., Prochnow A. (2019). Process Disturbances in Agricultural Biogas Production—Causes, mechanisms and effects on the biogas microbiome: A review. Energies.

[38] Alsouleman K., Linke B., Klang J., Klocke M., Krakat N., Theuerl S. (2016). Reorganisation of a mesophilic biogas microbiome as response to a stepwise increase of ammonium nitrogen induced by poultry manure supply. Bioresour. Technol..

[39] Klang J., Szewzyk U., Bock D., Theuerl S. (2019). Nexus between the microbial diversity level and the stress tolerance within the biogas process. Anaerobe.

[40] Westerholm M., Isaksson S., Karlsson Lindsjö O., Schnürer A. (2018). Microbial community adaptability to altered temperature conditions determines the potential for process optimisation in biogas production. Appl. Energy.

[41] Niu Q., Kubota K., Qiao W., Jing Z., Zhang Y., Yu-You L. (2014). Effect of ammonia inhibition on microbial community dynamic and process functional resilience in mesophilic methane fermentation of chicken manure. J. Chem. Technol. Biotechnol..

[42] Lv Z., Leite A.F., Harms H., Glaser K., Liebetrau J., Kleinsteuber S., Nikolausz M. (2019). Microbial community shifts in biogas reactors upon complete or partial ammonia inhibition. Appl. Microbiol. Biotechnol..

[43] Gutleben J., Chaib De Mares M., van Elsas J.D., Smidt H., Overmann J., Sipkema D. (2018). The multi-omics promise in context: From sequence to microbial isolate. Crit. Rev. Microbiol..

[44] Lloyd K.G., Steen A.D., Ladau J., Yin J., Crosby L. (2018). Phylogenetically novel uncultured microbial cells dominate earth microbiomes. mSystems.

[45] Schnürer A. (2013). Cristal ball—Know thy microorganism – why metagenomics is not enough!. Microb. Biotechnol..

[46] De Vrieze J., Ijaz U.Z., Saunders A.M., Theuerl S. (2018). Terminal restriction fragment length polymorphism is an “old school” reliable technique for swift microbial community screening in anaerobic digestion. Sci. Rep..

[47] Hassa J., Maus I., Off S., Pühler A., Scherer P., Klocke M., Schlüter A. (2018). Metagenome, metatranscriptome, and metaproteome approaches unraveled compositions and functional relationships of microbial communities residing in biogas plants. Appl. Microbiol. Biotechnol..

[48] Heyer R., Schallert K., Büdel A., Zoun R., Dorl S., Behne A., Kohrs F., Püttker S., Siewert C., Muth T. (2019). A Robust and universal metaproteomics workflow for research studies and routine diagnostics within 24 h using phenol extraction, FASP digest, and the MetaProteomeAnalyzer. Front. Microbiol..

[49] Ramette A. (2007). Multivariate analyses in microbial ecology. FEMS Microbiol. Ecol..

[50] Paliy O., Shankar V. (2016). Application of multivariate statistical techniques in microbial ecology. Mol. Ecol..

[51] King R., Baker M., Guntenspergen G. (2014). Use, misuse, and limitations of Threshold Indicator Taxa Analysis [TITAN] for natural resource management. Application of Threshold Concepts in Natural Resource Decision Makin.

[52] Berry D., Widder S. (2014). Deciphering microbial interactions and detecting keystone species with co-occurrence networks. Front. Microbiol..

[53] Karimi B., Maron P.A., Chemidlin-Prevost Boure N., Bernard N., Gilbert D., Ranjard L. (2017). Microbial diversity and ecological networks as indicators of environmental quality. Environ. Chem. Lett..

[54] Röttjers L., Faust K. (2018). From hairballs to hypotheses – biological insights from microbial networks. FEMS Microbiol. Rev..

[55] De Vrieze J., Saunders A.M., He Y., Fang J., Nielsen P.H., Verstraete W., Boon N. (2015). Ammonia and temperature determine potential clustering in the anaerobic digestion microbiome. Water Res..

[56] Mei R., Nobu M.K., Narihiro T., Kuroda K., Muñoz Sierra J., Wu Z., Ye L., Lee P.K.H., Lee P.H., van Lier J.B. (2017). Operation-driven heterogeneity and overlooked feed-associated populations in global anaerobic digester microbiome. Water Res..

[57] Sundberg C., Al-Soud W.A., Larsson M., Alm E., Yekta S.S., Svensson B.H., Sørensen S.J., Karlsson A. (2013). 454 pyrosequencing analyses of bacterial and archaeal richness in 21 full-scale biogas digesters. FEMS Microbiol. Ecol..

[58] Ziels R.M., Svensson B.H., Sundberg C., Larsson M., Karlsson A., Yekta S.S. (2018). Microbial rRNA gene expression and co-occurrence profiles associate with biokinetics and elemental composition in full-scale anaerobic digesters. Microb. Biotechnol..

[59] Faust K., Lahti L., Gonze D., de Vos W.M., Raes J. (2015). Metagenomics meets time series analysis: Unraveling microbial community dynamics. Curr. Opin. Microbiol..

[60] Shade A., Caporaso J.G., Handelsman J., Knight R., Fierer N. (2013). A meta-analysis of changes in bacterial and archaeal communities with time. ISME J..

[61] Calusinska M., Goux X., Fossépré M., Muller E.E.L., Wilmes P., Delfosse P. (2018). A year of monitoring 20 mesophilic full-scale bioreactors reveals the existence of stable but different core microbiomes in bio-waste and wastewater anaerobic digestion systems. Biotechnol. Biofuels.

[62] Jousset A., Bienhold C., Chatzinotas A., Gallien L., Gobet A., Kurm V., Kusel K., Rillig M.C., Rivett D.W., Salles J.F. (2017). Where less may be more: How the rare biosphere pulls ecosystems strings. ISME J..

[63] Werner J.J., Knights D., Garcia M.L., Scalfone N.B., Smith S., Yarasheski K., Cummings T.A., Beers A.R., Knight R., Angenent L.T. (2011). Bacterial community structures are unique and resilient in full-scale bioenergy systems. Proc. Natl. Acad. Sci. USA.

[64] Bonk F., Popp D., Weinrich S., Sträuber H., Kleinsteuber S., Harms H., Centler F. (2018). Intermittent fasting for microbes: How discontinuous feeding increases functional stability in anaerobic digestion. Biotechnol. Biofuels.

[65] Theuerl S., Klang J., Heiermann M., De Vrieze J. (2018). Marker microbiome clusters are determined by operational parameters and specific key taxa combinations in anaerobic digestion. Bioresour. Technol..

[66] Liebetrau J., Pfeiffer D., Thrän D. (2016). Collection of Methods for Biogas—Methods to Determine Parameters for Analysis Purposes and Parameters that Describe Processes in the Biogas Sector.

[67] Hansen K.H., Angelidaki I., Ahring B.K. (1998). Anaerobic digestion of swine manure: Inhibition by ammonia. Water Res..

[68] (2016). VDI 4630—Fermentation of Organic Materials—Characterization of the Substrate, Sampling, Collection of Material Data, Fermentation Tests.

[69] Takahashi S., Tomita J., Nishioka K., Hisada T., Nishijima M. (2014). Development of a prokaryotic universal primer for simultaneous analysis of bacteria and archaea using next-generation sequencing. PLoS ONE.

[70] Andrews S. (2010). FastQC: A quality control tool for high throughput sequence data. http://www.bioinformatics.babraham.ac.uk/projects/fastqc.

[71] Magoč T., Salzberg S.L. (2011). FLASH: Fast length adjustment of short reads to improve genome assemblies. Bioinformatics.

[72] Martin M. (2011). Cutadapt removes adapter sequences from high-throughput sequencing reads. EMBnet J..

[73] Joshi N.A., Fass J.N. (2011). Sickle: A Sliding-Window, Adaptive, Quality-Based Trimming Tool for FastQ Files [version 1.33]. https://github.com/najoshi/sickle.

[74] Caporaso J.G., Kuczynski J., Stombaugh J., Bittinger K., Bushman F.D., Costello E.K., Fierer N., Gonzalez Peña A., Goodrich J.K., Gordon J.I. (2010). QIIME allows analysis of high-throughput community sequencing data. Nat. Methods.

[75] Bolyen E., Rideout J.R., Dillon M.R., Bokulich N.A., Abnet C.C., Al-Ghalith G.A., Alexander H., Alm E.J., Arumugam M., Asnicar F. (2019). Reproducible, interactive, scalable, and extensible microbiome data science using QIIME 2. Nat. Biotechnol..

[76] Zhang L., Loh K.-C., Lim J.W., Zhang J. (2019). Bioinformatics analysis of metagenomics data of biogas-producing microbial communities in anaerobic digesters: A review. Renew. Sustain. Energy Rev..

[77] Edgar R.C. (2010). Search and clustering orders of magnitude faster than BLAST. Bioinformatics.

[78] Klang J., Theuerl S., Szewzyk U., Huth M., Tölle R., Klocke M. (2015). Dynamic variation of the microbial community structure during the long-time mono-fermentation of maize and sugar beet silage. Microb. Biotechnol..

[79] Rademacher A., Nolte C., Schönberg M., Klocke M. (2012). Temperature increases from 55 to 75 °C in a two-phase biogas reactor result in fundamental alterations within the bacterial and archaeal community structure. Appl. Microbiol. Biotechnol..

[80] (2018). R Core Team R: A language and environment for statistical computing. https://www.r-project.org/.

[81] Oksanen J., Blanchet F.G., Friendly M., Kindt R., Legendre P., McGlinn D., Minchin P.R., O’Hara R.B., Simpson G.L., Solymos P. vegan: Community Ecology Package 2018. https://github.com/vegandevs/vegan.

[82] Clarke K.R. (1993). Non-parametic multivariate analyses of changes in community structure. Aust. J. Ecol..

[83] Bray J.R., Curtis J.T. (1957). An Ordination of the Upland Forest Communities of Southern Wisconsin. Ecol. Monogr..

[84] Baker M.E., King R.S. (2010). A new method for detecting and interpreting biodiversity and ecological community thresholds. Methods Ecol. Evol..

[85] Carney R.L. (2019). Microbial community dynamics within impacted coastal ecosystems. Ph.D. Thesis.

[86] Simonin M., Voss K.A., Hassett B.A., Rocca J.D., Wang S.-Y., Bier R.L., Violin C.R., Wright J.P., Bernhardt E.S. (2019). In search of microbial indicator taxa: Shifts in stream bacterial communities along an urbanization gradient. Environ. Microbiol..

[87] Aschonitis V., Karydas C.G., Iatrou M., Mourelatos S., Metaxa I., Tziachris P., Iatrou G. (2019). An integrated approach to assessing the soil quality and nutritional status of large and long-term cultivated rice agro-ecosystems. Agriculture.

[88] Kim J., Lee C. (2016). Response of a continuous anaerobic digester to temperature transitions: A critical range for restructuring the microbial community structure and function. Water Res..

[89] Luo G., De Francisci D., Kougias P.G., Treu L., Zhu X., Angelidaki I. (2015). New steady-state microbial community compositions and process performances in biogas reactors induced by temperature disturbances. Biotechnol. Biofuels.

[90] Weiland P. (2010). Biogas production: Current state and perspectives. Appl. Microbiol. Biotechnol..

[91] Li Y.Q., Liu C.M., Wachemo A.C., Li X.J. (2018). Effects of liquid fraction of digestate recirculation on system performance and microbial community structure during serial anaerobic digestion of completely stirred tank reactors for corn stover. Energy.

[92] Zamanzadeh M., Hagen L.H., Svensson K., Linjordet R., Horn S.J. (2016). Anaerobic digestion of food waste e Effect of recirculation and temperature on performance and microbiology. Water Res..

[93] Maus I., Kim Y.S., Wibberg D., Stolze Y., Off S., Antonczyk S., Pühler A., Scherer P., Schlüter A. (2017). Biphasic study to characterize agricultural biogas plants by high-throughput 16S rRNA gene amplicon sequencing and microscopic analysis. J. Microbiol. Biotechnol..

[94] Nobu M.K., Dodsworth J.A., Murugapiran S.K., Rinke C., Gies E.A., Webster G., Schwientek P., Kille P., Parkes J.R., Sass H. (2016). Phylogeny and physiology of candidate phylum ‘*Atribacteria’* (OP9/JS1) inferred from cultivation independent genomics. ISME J..

[95] Leng L., Yang P., Singh S., Zhuang H., Xu L., Chen W.-H., Dolfing J., Li D., Zhang Y., Zeng H. (2018). A review on the bioenergetics of anaerobic microbial metabolism close to the thermodynamic limits and its implications for digestion applications. Bioresour. Technol..

[96] Morris B.E.L., Henneberger R., Huber H., Moissl-Eichinger C. (2013). Microbial syntrophy: Interaction for the common good. FEMS Microbiol. Rev..

[97] Schnürer A., Nordberg A. (2008). Ammonia, a selective agent for methane production by syntrophic acetate oxidation at mesophilic temperature. Water Sci. Technol..

[98] De Vrieze J., Hennebel T., Boon N., Verstraete W. (2012). *Methanosarcina*: The rediscovered methanogen for heavy duty biomethanation. Bioresour. Technol..

[99] Lewin G.R., Carlos C., Chevrette M.G., Horn H.A., McDonald B.R., Stankey R.J., Fox B.G., Currie C.R. (2016). Evolution and ecology of *Actinobacteria* and their bioenergy applications. Annu. Rev. Microbiol..

[100] Wang C., Dong D., Wang H., Müller K., Qin Y., Wang H., Wu W. (2016). Metagenomic analysis of microbial consortia enriched from compost: New insights into the role of *Actinobacteria* in lignocellulose decomposition. Biotechnol. Biofuels.

[101] Hülsemann B., Zhou L., Merkle W., Hassa J., Müller J., Oechsner H. (2020). Biomethane potential test: Influence of inoculum and the digestion system. Appl. Sci..

[102] Allison S.D., Martiny J.B. (2008). Resistance, resilience, and redundancy in microbial communities. Proc. Natl. Acad. Sci. USA.

[103] Drosg B., Frost P., Baxter D. (2013). Process monitoring in biogas plants. IEA Bioenergy Task 37—Energy from Biogas.

