# Supplementary material for: Microbiome Diversity and Community-Level Change Points within Manure-based small Biogas Plants
Source: Microorganisms. 2020 Aug 1;8(8):1169. doi: 10.3390/microorganisms8081169 (PMC7464807; doi:10.3390/microorganisms8081169)
Supplement: Supplementary file 1 [file microorganisms-08-01169-s001.zip › SupplementFigures - SmallManurePlants - Theuerl.pdf]

Supplement - Figures

Manuscript title: `Microbiome Diversity and Community-Level Change Points within Manure-based small Biogas Plants ´

Susanne Theuerl [1*], Johanna Klang [1], Benedikt Hülsemann [2], Torsten Mächtig [3], Julia Hassa [1,4]

[1] Leibniz Institute for Agricultural Engineering and Bioeconomy, Max-Eyth-Allee 100, 14469 Potsdam, Germany; stheuerl@atb-potsdam.de (ST), jklang@atb-potsdam.de (JK), jhassa@atb-potsdam.de (JH)
[2] University of Hohenheim, The State Institute of Agricultural Engineering and Bioenergy, Garbenstraße 9, 70599 Stuttgart, Germany; Benedikt.Huelsemann@uni-hohenheim.de (BH)
[3] Kiel University, Institute of Agricultural Engineering, Olshausenstraße 40, 24098 Kiel, Germany; tstefan@ilv.uni-kiel.de (TM)
[4] Bielefeld University, Center for Biotechnology (CeBiTec), Universitätsstr. 27, 33615 Bielefeld, Germany; jhassa@CeBiTec.Uni-Bielefeld.de (JH)
* Correspondence: susanne.theuerl@googlemail.com; Tel.: +49-331-5699-900

On the following pages all supplementary figures mentioned in the main manuscript are provided.

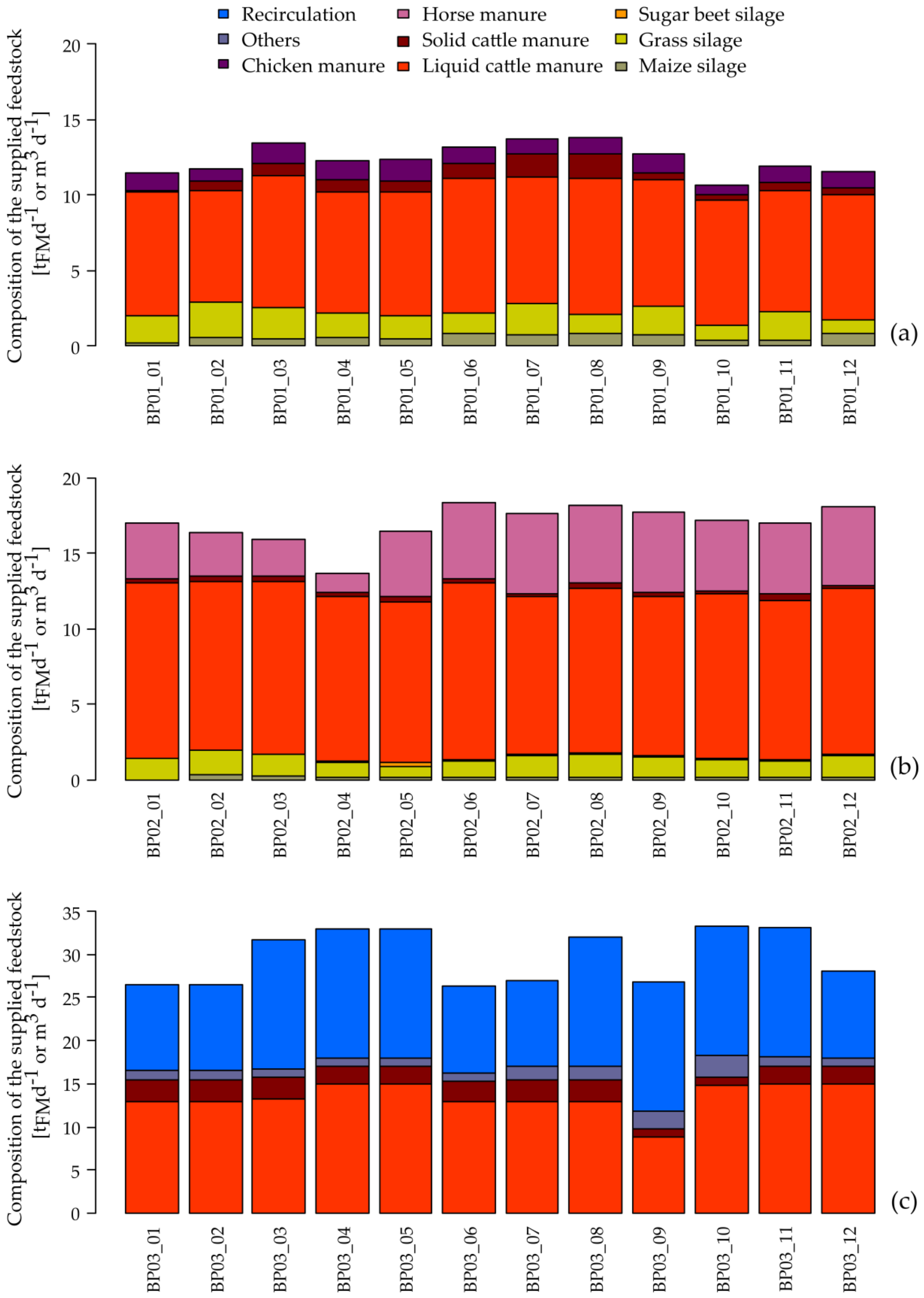

Figure S1. Feedstock composition of the analyzed manure-based small biogas plants BP 01 (a), BP 02 (b) and BP 03 (c) over a time period of one year.

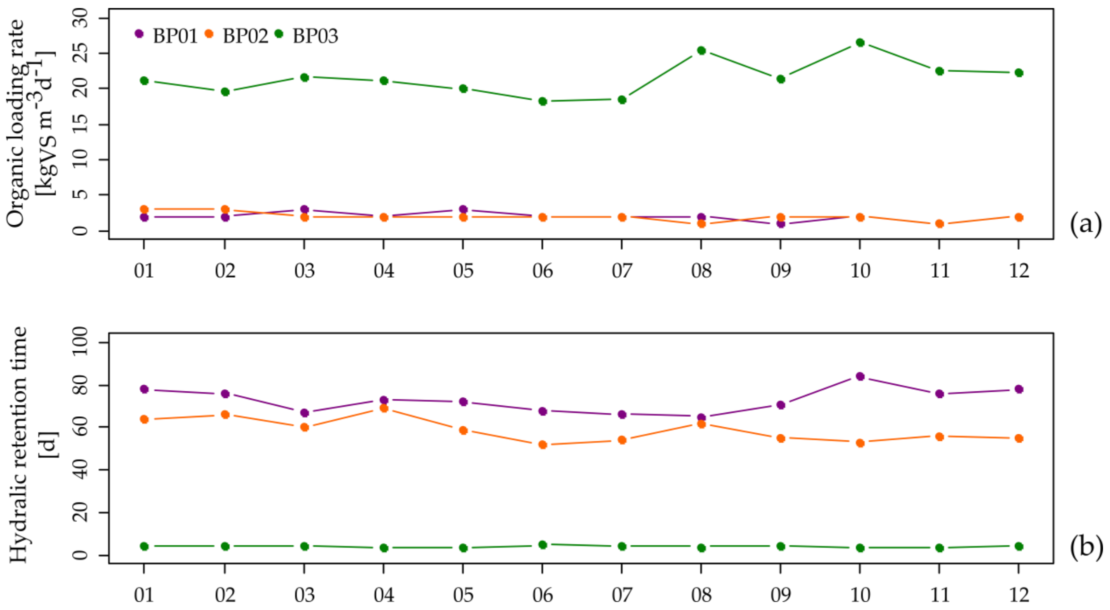

Figure S2. Organic loading rate (a) and the corresponding hydraulic retention time (b) of the analyzed manure-based small biogas plants over a time period of one year.

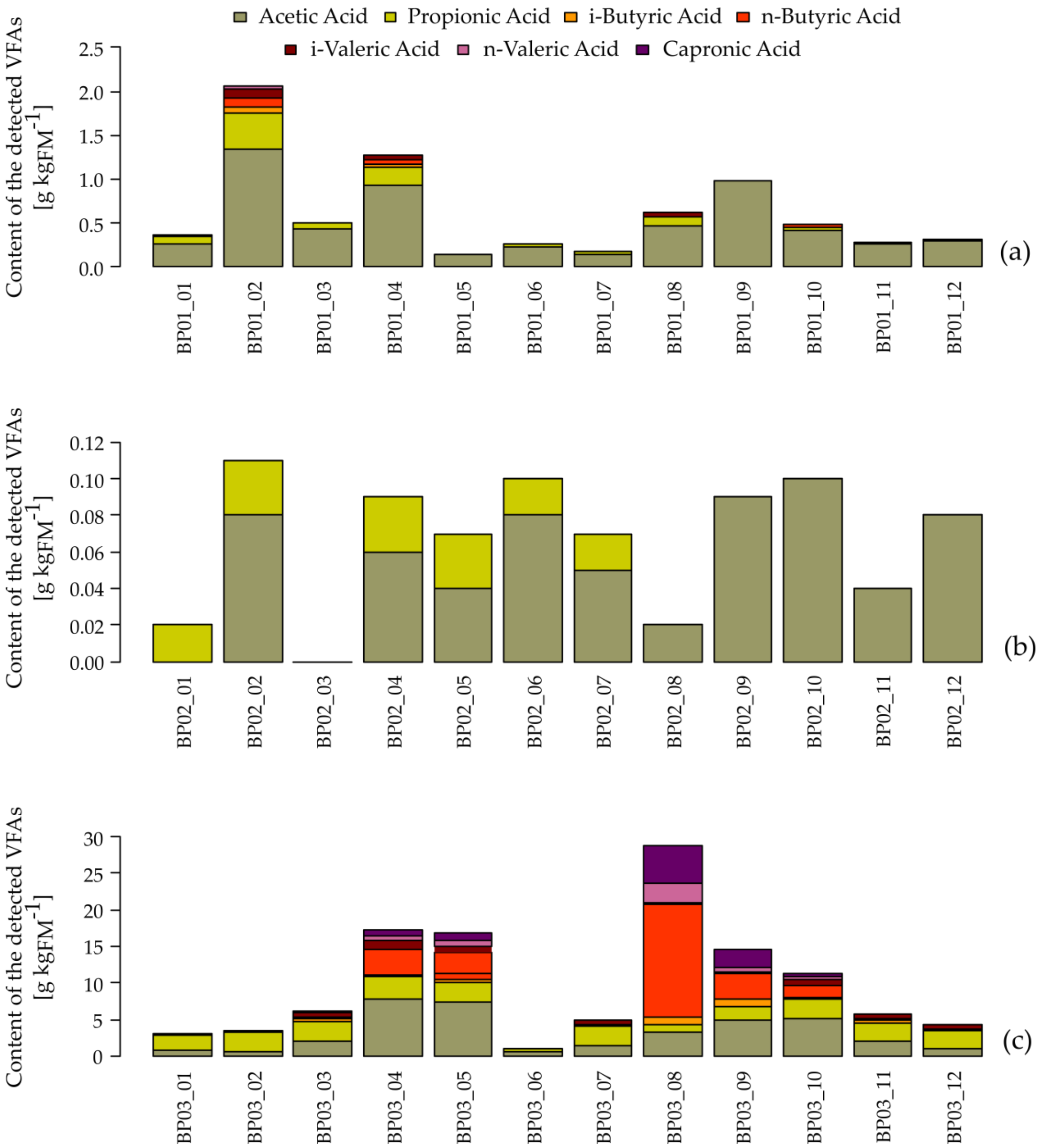

Figure S3. Recorded volatile fatty acid spectrum of the analyzed manure-based small biogas plants BP 01 (a), BP 02 (b) and BP 03 (c) over a time period of one year. To be considered: different axis scaling.

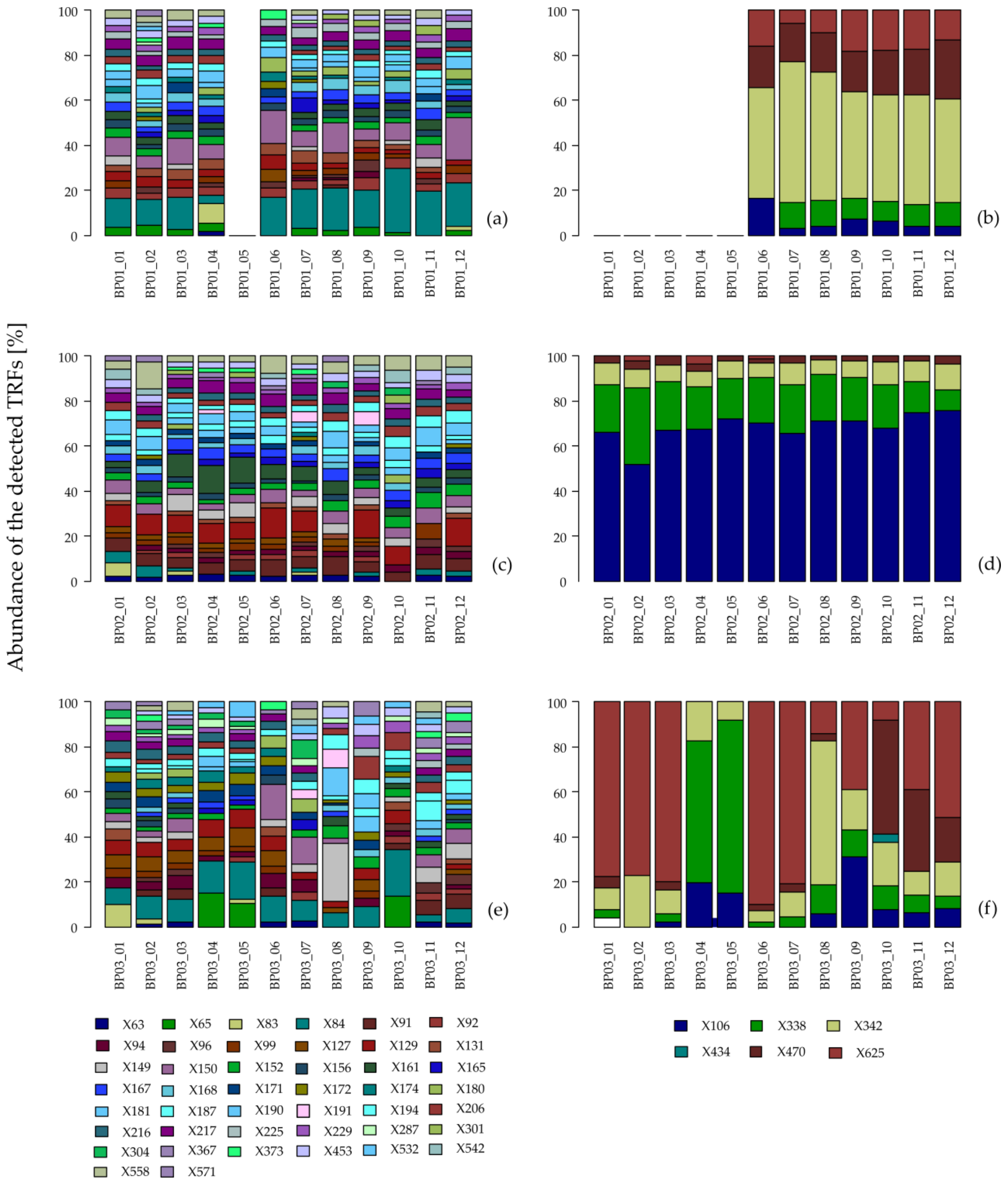

Figure S4. Distribution diagrams of the recorded bacterial (a, c, e) and archaeal (b, d, f) terminal restriction fragment (TRFs) based on their relative abundance within the three investigated manure-based small biogas plants BP 01 (a, b), BP 02 (c, d) and BP 03 (e, f).

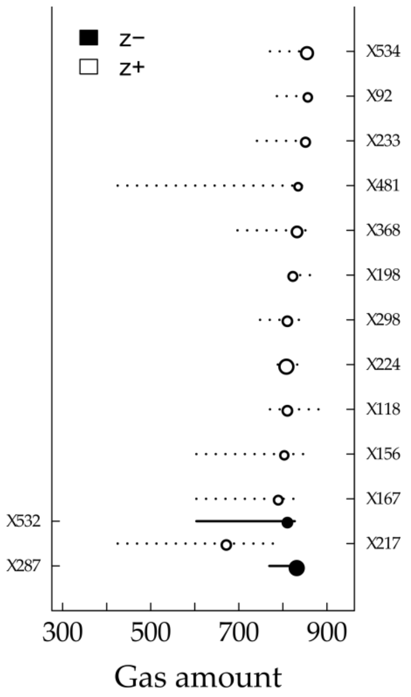

Figure S5. Threshold Indicator Taxa Analysis (TITAN) on the individual density of detected terminal restriction fragments (TFRs) in response to the amount of produced biogas. Circles represent the change points of indicative TRFs that decreased (black, negative response, left y-axis) or increased (white, positive response, right y-axis) with increasing `environmental´ gradients and are sized based on the magnitude of the response (z-score; the larger the circle, the stronger the response). Horizontal lines represent 5th and 95th quantiles of 500 bootstrap replicates. Warning message from TITAN has to be considered:: `low number of pure and reliable taxa, sum (z) output should be interpreted with caution´
