# Supplementary material for: Microbiome Diversity and Community-Level Change Points within Manure-based small Biogas Plants
Source: Microorganisms. 2020 Aug 1;8(8):1169. doi: 10.3390/microorganisms8081169 (PMC7464807; doi:10.3390/microorganisms8081169)
Supplement: Supplementary file 1 [file microorganisms-08-01169-s001.zip › SupplementTables - SmallManurePlants - Theuerl.pdf]

Supplement - Tables

Manuscript title: `Microbiome Diversity and Community-Level Change Points within Manure-based small Biogas Plants ´

Susanne Theuerl [1*], Johanna Klang [1], Benedikt Hülsemann [2], Torsten Mächtig [3], Julia Hassa [1,4]

[1] Leibniz Institute for Agricultural Engineering and Bioeconomy, Max-Eyth-Allee 100, 14469 Potsdam, Germany; stheuerl@atb-potsdam.de (ST), jklang@atb-potsdam.de (JK), jhassa@atb-potsdam.de (JH)

[2] University of Hohenheim, The State Institute of Agricultural Engineering and Bioenergy, Garbenstraße 9, 70599 Stuttgart, Germany; Benedikt.Huelsemann@uni-hohenheim.de (BH)

[3] Kiel University, Institute of Agricultural Engineering, Olshausenstraße 40, 24098 Kiel, Germany; tstefan@ilv.uni-kiel.de (TM)

[4] Bielefeld University, Center for Biotechnology (CeBiTec), Universitätsstr. 27, 33615 Bielefeld, Germany; jhassa@CeBiTec.Uni-Bielefeld.de (JH)

* Correspondence: susanne.theuerl@googlemail.com; Tel.: +49-331-5699-900

On the following pages all supplementary tables mentioned in the main manuscript are provided.

Table S1. Summary of process engineering and process chemical parameters of the main anaerobic digesters of the analyzed manure-based small biogas plants over the entire sampling period. FM = fresh mass, OLR = organic loading rate, HRT = hydraulic retention time, ns = not supplied, TS = total solids, VS = volatile solids, TAN = total ammonium nitrogen, NH_3 = free ammonia nitrogen, HAc = acetic acid, HPr = propionic acid, HiB = iso-butyric acid, HnB, n-butyric acid, HiV = iso-valeric acid, HnV = n-valeric acid, HC = capronic acid, VFA = volatile fatty acids, CH_4 = methane.

Biogas plant	Sampling	Supplied feedstocks [t_{FM} d^{-1}]									OLR [kg_{VS} m^{-3} d^{-1}]	HRT [d]	Temperature [°C]
		**Maize silage**	**Grass silage**	**Sugar beet silage**	**Liquid cattle manure**	**Solid cattle manure**	**Chicken manure**	**Horse manure**	**Others**	**Recirculate**			
	Sep 17	0.2	1.8		8.2	0.1	1.2				1.7	77.6	42.1
	Oct 17	0.6	2.3		7.4	0.6	0.8				2.2	76.4	42.0
	Nov 17	0.5	2.0		8.8	0.8	1.4				2.6	66.5	41.6
	Dec 17	0.6	1.6		8.0	0.8	1.3				1.9	73.0	41.8
	Jan 18	0.5	1.5		8.2	0.7	1.5				2.5	71.8	41.9
BP 01	Feb 18	0.8	1.4	ns	8.9	1.0	1.1	ns	ns	ns	2.1	68.0	41.9
	Mar 18	0.7	2.1		8.4	1.5	1.0				2.2	65.6	41.9
	Apr 18	0.8	1.3		9.0	1.6	1.1				2.3	65.1	42.0
	May 18	0.7	1.9		8.4	0.5	1.2				1.5	70.9	41.9
	Jun 18	0.4	1.0		8.3	0.3	0.7				1.9	84.0	42.1
	Jul 18	0.4	1.9		8.0	0.5	1.1				1.4	75.8	42.0
	Aug 18	0.8	0.9		8.3	0.5	1.1				1.7	78.0	42.0
	Sep 17	0.0	1.4	0.0	11.6	0.3		3.7			2.7	64.4	41.9
	Oct 17	0.3	1.6	0.0	11.2	0.4		2.9			2.6	65.5	41.9
	Nov 17	0.2	1.5	0.0	11.4	0.4		2.4			1.8	59.6	41.9
	Dec 17	0.1	1.0	0.1	10.9	0.3		1.3			1.5	69.3	41.9
	Jan 18	0.1	0.8	0.2	10.7	0.3		4.4			2.1	58.5	41.9
BP 02	Feb 18	0.1	1.1	0.1	11.7	0.3	ns	5.1	ns	ns	1.9	52.0	41.9
	Mar 18	0.1	1.5	0.1	10.4	0.2		5.3			2.2	54.1	41.9
	Apr 18	0.1	1.6	0.1	10.9	0.3		5.2			1.3	62.4	41.9
	May 18	0.1	1.4	0.1	10.5	0.3		5.3			2.3	54.5	41.9
	Jun 18	0.1	1.2	0.1	10.9	0.2		4.7			2.0	53.4	41.9
	Jul 18	0.1	1.1	0.1	10.6	0.4		4.7			1.0	56.3	41.9
	Aug 18	0.1	1.5	0.1	11.0	0.2		5.2			1.6	54.6	41.9
	Aug 17				13.0	2.5			1.0	10.0	21.3	4.5	52.0
	Sep 17				13.0	2.5			1.0	10.0	19.6	4.5	52.0
	Oct 17				13.2	2.5			1.0	15.0	21.6	3.8	51.6
	Nov 17				15.0	2.0			1.0	15.0	21.2	3.6	42.5
	Dec 17				15.0	2.0			1.0	15.0	20.0	3.6	42.5
BP 03	Jan 18	ns	ns	ns	13.0	2.3	ns	ns	1.0	10.0	18.3	4.6	46.3
	Feb 18				13.0	2.5			1.5	10.0	18.5	4.4	49.5
	Mar 18				13.0	2.5			1.5	15.0	25.4	3.7	49.5
	Apr 18				8.8	1.0			2.0	15.0	21.4	4.5	44.3
	May 18				14.8	0.9			2.6	15.0	26.6	3.6	42.5
	Jun 18				15.0	2.0			1.1	15.0	22.5	3.6	42.5
	Jul 18				15.0	2.0			1.0	10.0	22.4	4.3	42.5

Table S1. Continued.

Biogas plant	Sampling	TS [% FM]	VS [% TS]	pH [-]	TAN [g L^{-1}]	NH$_3$ [mg L^{-1}]	HAc [g L^{-1}]	HPr [g L^{-1}]	HiB [g L^{-1}]	HnB [g L^{-1}]	HiV [g L^{-1}]	HnV [g L^{-1}]	HC [g L^{-1}]	VFA [g L^{-1}]	Calculated biogas amount [m^3_N d^{-1}]	CH$_4$ [%]
BP 01	Sep 17	11.5	68.3	8.3	2.2	576	0.3	0.1	0.0	0.0	0.0	0.0	0.0	0.3	900.1	58.0
	Oct 17	7.9	73.1	8.0	4.5	708	1.3	0.3	0.0	0.1	0.1	0.0	0.0	1.9	869.2	
	Nov 17	11.5	67.9	8.3	7.3	1721	0.4	0.1	0.0	0.0	0.0	0.0	0.0	0.5	864.4	
	Dec 17	10.3	68.9	8.1	3.9	729	0.9	0.2	0.0	0.0	0.0	0.0	0.0	1.2	838.8	
	Jan 18	9.9	69.7	8.4	6.4	2103	0.1	0.0	0.0	0.0	0.0	0.0	0.0	0.1	892.7	
	Feb 18	10.2	70.3	8.2	6.0	1413	0.2	0.0	0.0	0.0	0.0	0.0	0.0	0.3	890.2	
	Mar 18	9.5	70.1	8.1	6.5	1231	0.1	0.0	0.0	0.0	0.0	0.0	0.0	0.2	837.9	
	Apr 18	10.1	68.2	8.1	3.9	676	0.5	0.1	0.0	0.0	0.0	0.0	0.0	0.6	892.3	
	May 18	11.3	68.6	8.1	6.0	1109	1.0	0.0	0.0	0.0	0.0	0.0	0.0	1.0	658.3	
	Jun 18	10.8	66.6	8.1	6.2	1050	0.4	0.0	0.0	0.0	0.0	0.0	0.0	0.5	836.7	
	Jul 18	11.2	69.3	8.4	6.5	2001	0.3	0.0	0.0	0.0	0.0	0.0	0.0	0.3	865.7	
	Aug 18	12.7	70.1	8.2	7.1	1620	0.3	0.0	0.0	0.0	0.0	0.0	0.0	0.3	864.0	
BG 02	Sep 17	8.2	70.6	7.5	2.1	114.6	0.0	0.0	0.0	0.0	0.0	0.0	0.0	0.0	776.3	55.0
	Oct 17	8.9	69.8	7.5	2.2	104.9	0.1	0.0	0.0	0.0	0.0	0.0	0.0	0.1	901.1	
	Nov 17	9.8	69.9	7.5	2.3	113.6	0.0	0.0	0.0	0.0	0.0	0.0	0.0	0.0	694.0	
	Dec 17	9.3	69.1	7.6	1.7	116.6	0.1	0.0	0.0	0.0	0.0	0.0	0.0	0.1	841.5	
	Jan 18	9.0	70.6	7.5	2.5	125.8	0.0	0.0	0.0	0.0	0.0	0.0	0.0	0.1	855.3	
	Feb 18	8.6	69.2	7.5	2.7	141.1	0.1	0.0	0.0	0.0	0.0	0.0	0.0	0.1	856.2	
	Mar 18	9.0	70.3	7.7	2.6	206.6	0.0	0.0	0.0	0.0	0.0	0.0	0.0	0.1	844.9	
	Apr 18	9.0	69.0	7.6	1.9	123.5	0.0	0.0	0.0	0.0	0.0	0.0	0.0	0.0	851.1	
	May 18	8.5	66.6	7.6	2.8	185.2	0.1	0.0	0.0	0.0	0.0	0.0	0.0	0.1	844.0	
	Jun 18	7.9	66.8	7.5	2.6	142.1	0.1	0.0	0.0	0.0	0.0	0.0	0.0	0.1	799.1	
	Jul 18	7.6	68.6	7.6	2.9	204.0	0.0	0.0	0.0	0.0	0.0	0.0	0.0	0.0	817.2	
	Aug 18	7.7	68.0	7.7	3.1	255.7	0.1	0.0	0.0	0.0	0.0	0.0	0.0	0.1	857.3	
BG 03	Aug 17	7.6	78.4	7.7	1.3	196.2	0.8	1.7	0.0	0.0	0.1	0.0	0.0	2.6	785.4	51.0
	Sep 17	8.4	75.9	7.7	1.8	248.5	0.6	2.2	0.0	0.0	0.1	0.0	0.0	2.9	788.5	
	Oct 17	9.9	75.0	7.4	1.6	131.8	2.0	2.2	0.3	0.2	0.4	0.2	0.0	5.2	812.6	
	Nov 17	9.8	77.6	6.1	2.1	4.6	7.8	2.5	0.2	2.3	0.7	0.4	0.4	14.3	720.1	
	Dec 17	9.6	78.4	6.3	2.2	8.0	7.5	2.2	0.3	2.5	0.5	0.5	0.6	13.9	801.9	
	Jan 18	7.6	72.2	8.1	2.1	441.3	0.7	0.3	0.0	0.0	0.0	0.0	0.0	0.9	700.0	
	Feb 18	8.3	73.8	8.0	2.0	414.2	1.4	2.3	0.1	0.0	0.4	0.0	0.0	4.1	549.0	
	Mar 18	9.8	84.2	5.2	1.6	0.8	3.3	0.8	0.8	10.4	0.2	1.5	2.7	19.6	301.0	
	Apr 18	12.9	83.9	kA	1.6	4.1	5.0	1.5	0.7	2.3	0.1	0.4	1.3	11.3	201.4	
	May 18	11.8	80.1	6.9	2.1	29.2	5.0	2.2	0.2	1.1	0.5	0.2	0.2	9.5	151.3	
	Jun 18	6.8	74.3	7.6	2.1	139.1	2.1	2.0	0.3	0.1	0.3	0.0	0.0	4.8	765.1	
	Jul 18	7.0	75.1	7.5	1.8	98.6	1.0	2.0	0.2	0.0	0.3	0.0	0.0	3.5	762.2	

Table S2. Taxonomic profiles at the phylum and genus level of the main anaerobic digesters of the analyzed manure-based small biogas plants after a three month lasting lead time.

	**Domain**	**Phylum**	**BP 01**	**BP 02**	**BP03**
**Taxa > 1%**	*Bacteria*	*Actinobacteria*	19.14	3.38	14.20
	Bacteria	*Atribacteria*	1.10	1.21	0.15
	Bacteria	*Bacteroidetes*	0.11	12.30	10.36
	Bacteria	*Chloroflexi*	1.09	0.51	0.45
	Bacteria	*Cloacimonetes*	0.00	11.49	0.02
	Archaea	*Euryarchaeota*	7.95	10.05	7.72
	Bacteria	*Fibrobacteres*	0.15	1.60	0.01
	Bacteria	*Firmicutes*	65.60	52.64	56.50
	Bacteria	*Patescibacteria*	0.17	1.38	1.17
	Bacteria	*Proteobacteria*	3.68	1.44	4.65
	Bacteria	*Spirochaetes*	0.00	1.18	0.13
	Bacteria	*Tenericutes*	0.01	1.27	0.23
	Unassigned	*Other*	0.01	0.16	3.31
		Taxa < 1%	0.99	1.37	1.10
**Taxa < 1%**	*Bacteria*	*Acidobacteria*	0.00	0.12	0.01
	Bacteria	*Armatimonadetes*	0.00	0.05	0.09
	Bacteria	*BRC1*	0.03	0.03	0.01
	Archaea	*Crenarchaeota*	0.00	0.12	0.00
	Bacteria	*Cyanobacteria*	0.02	0.03	0.00
	Bacteria	*Deinococcus-Thermus*	0.07	0.04	0.03
	Bacteria	*Gemmatimonadetes*	0.16	0.04	0.02
	Bacteria	*Halanaerobiaeota*	0.07	0.01	0.01
	Bacteria	*Hydrogenedentes*	0.00	0.07	0.01
	Bacteria	*Kiritimatiellaeota*	0.00	0.02	0.00
	Bacteria	*Planctomycetes*	0.09	0.08	0.05
	Bacteria	*Synergistetes*	0.46	0.59	0.82
	Bacteria	*Thermotogae*	0.00	0.00	0.03
	Bacteria	*Verrucomicrobia*	0.08	0.15	0.02
	Bacteria	*WPS-2*	0.01	0.02	0.01
	Bacteria	*WS4*	0.00	0.01	0.00
**proportions of distribution**	*Bacteria*		92.0	89.7	89.0
	Archaea		7.9	10.2	7.7
	Unassigned		0.0	0.2	3.3

Table S2. Continued.

Domain	Phylum	Class	Order	Family	Genus	BP 01	BP 02	BP 03
Bacteria	*Actinobacteria*	*Actinobacteria*	*Corynebacteriales*	*Corynebacteriaceae*	*Corynebacterium 1*	5.64	0.83	4.16
Bacteria	*Actinobacteria*	*Actinobacteria*	*Micrococcales*	*Dermabacteraceae*	*Brachybacterium*	3.12	0.06	0.27
Bacteria	*Bacteroidetes*	*Bacteroidia*	*Bacteroidales*	*Dysgonomonadaceae*	*Proteiniphilum*	0.07	0.27	1.70
Bacteria	*Bacteroidetes*	*Bacteroidia*	*Bacteroidales*	*Marinilabiliaceae*	*Ruminofilibacter*	0.00	4.63	0.01
Bacteria	*Bacteroidetes*	*Bacteroidia*	*Bacteroidales*	*Rikenellaceae*	*Rikenellaceae RC9 gut group*	0.00	1.02	4.90
Bacteria	*Cloacimonetes*	*Cloacimonadia*	*Cloacimonadales*	*Cloacimonadaceae*	*Cloacimonadaceae W5*	0.00	11.11	0.01
Archaea	*Euryarchaeota*	*Methanobacteria*	*Methanobacteriales*	*Methanobacteriaceae*	*Methanobrevibacter*	6.34	1.42	7.13
Archaea	*Euryarchaeota*	*Methanomicrobia*	*Methanosarcinales*	*Methanosaetaceae*	*Methanosaeta*	0.00	7.83	0.13
Bacteria	*Firmicutes*	*Bacilli*	*Bacillales*	*Bacillaceae*	*Bacillus*	2.61	0.37	0.56
Bacteria	*Firmicutes*	*Bacilli*	*Lactobacillales*	*Aerococcaceae*	*Facklamia*	1.50	0.07	0.16
Bacteria	*Firmicutes*	*Bacilli*	*Lactobacillales*	*Carnobacteriaceae*	*Atopostipes*	2.86	0.15	0.02
Bacteria	*Firmicutes*	*Bacilli*	*Lactobacillales*	*Carnobacteriaceae*	*Jeotgalibaca*	1.81	0.04	0.09
Bacteria	*Firmicutes*	*Bacilli*	*Lactobacillales*	*Carnobacteriaceae*	*Unc Carnobacteriaceae*	2.72	0.50	1.38
Bacteria	*Firmicutes*	*Clostridia*	*Clostridiales*	*Caldicoprobacteraceae*	*Caldicoprobacter*	1.04	5.06	0.03
Bacteria	*Firmicutes*	*Clostridia*	*Clostridiales*	*Clostridiaceae 1*	*Clostridium sensu stricto 1*	1.87	0.46	1.28
Bacteria	*Firmicutes*	*Clostridia*	*Clostridiales*	*Family XI*	*Gallicola*	1.91	0.02	0.22
Bacteria	*Firmicutes*	*Clostridia*	*Clostridiales*	*Family XI*	*Sedimentibacter*	0.06	4.59	0.06
Bacteria	*Firmicutes*	*Clostridia*	*Clostridiales*	*Family XI*	*Tepidimicrobium*	1.60	0.01	0.01
Bacteria	*Firmicutes*	*Clostridia*	*Clostridiales*	*Family XIII*	*[Eubacterium] nodatum group*	0.12	0.04	1.93
Bacteria	*Firmicutes*	*Clostridia*	*Clostridiales*	*Lachnospiraceae*	*Herbinix*	0.14	2.03	0.10
Bacteria	*Firmicutes*	*Clostridia*	*Clostridiales*	*Lachnospiraceae*	*Lachnospiraceae NK3A20 group*	3.22	1.25	1.92
Bacteria	*Firmicutes*	*Clostridia*	*Clostridiales*	*Lachnospiraceae*	*Syntrophococcus*	0.00	0.00	2.66
Bacteria	*Firmicutes*	*Clostridia*	*Clostridiales*	*Lachnospiraceae*	*Unc Lachnospiraceae*	0.06	0.04	2.08
Bacteria	*Firmicutes*	*Clostridia*	*Clostridiales*	*Peptostreptococcaceae*	*ambigous Peptostreptococcaceae*	1.06	0.39	1.54
Bacteria	*Firmicutes*	*Clostridia*	*Clostridiales*	*Peptostreptococcaceae*	*Paeniclostridium*	3.32	1.89	5.06
Bacteria	*Firmicutes*	*Clostridia*	*Clostridiales*	*Peptostreptococcaceae*	*Unc Peptostreptococcaceae*	4.81	2.33	6.74
Bacteria	*Firmicutes*	*Clostridia*	*Clostridiales*	*Ruminococcaceae*	*Fastidiosipila*	4.40	0.85	1.14
Bacteria	*Firmicutes*	*Clostridia*	*Clostridiales*	*Ruminococcaceae*	*Ruminiclostridium*	0.18	4.70	0.09
Bacteria	*Firmicutes*	*Clostridia*	*Clostridiales*	*Ruminococcaceae*	*Ruminiclostridium 1*	0.11	1.80	0.05
Bacteria	*Firmicutes*	*Clostridia*	*Clostridiales*	*Ruminococcaceae*	*Ruminococcus 1*	0.01	0.09	1.78
Bacteria	*Firmicutes*	*Clostridia*	*Clostridiales*	*Ruminococcaceae*	*Saccharofermentans*	0.02	0.00	1.57
Bacteria	*Firmicutes*	*Clostridia*	*Clostridiales*	*Ruminococcaceae*	*Unc Ruminococcaceae*	0.14	1.57	0.39
Bacteria	*Firmicutes*	*Clostridia*	*Clostridiales*	*Syntrophomonadaceae*	*Unc Syntrophomonadaceae*	5.44	2.06	0.02
Bacteria	*Firmicutes*	*Clostridia*	*MBA03*	*uncultured bacterium*	*Unc Clostridia MBA03*	0.02	4.49	0.01
Bacteria	*Firmicutes*	*Erysipelotrichia*	*Erysipelotrichales*	*Erysipelotrichaceae*	*Turicibacter*	3.32	1.05	2.49
Bacteria	*Firmicutes*	*Negativicutes*	*Selenomonadales*	*Acidaminococcaceae*	*Succiniclasticum*	0.00	0.00	1.72
					<1,5%	31.19	16.98	27.14
					Unclassified	9.27	19.98	19.47

Table S3. Community-level change points (ComCP) for selected operational and chemical process parameters based on the performed Threshold Indicator Taxa Analysis (TITAN) for negative (fsum(z-)) and positive (fsum(z+)) responders using only those taxa (terminal restriction fragment, TRFs) that are determined to be pure and reliable indicators. LCM = liquid cattle manure, TAN = total ammonium nitrogen, NH_3 = ammonia nitrogen, VFA = volatile fatty acids, HAc = acetic acid, HPr = propionic acid.

Environmental gradient	fsum(z-) ComCP	$ComCP_{min}$	$ComCP_{max}$	fsum(z+) ComCP	$ComCP_{min}$	$ComCP_{max}$	Intersect point
Grass silage	2.35	0.00	6,55	7.20	5.60	11.40	6.75
LCM	49.3	49,3	59,9	56.3	49.1	60,2	50.7
TAN	2.20	2.09	3.36	3.00	2.78	3.09	2.3
NH_3	331	193	493	625	412	642	193
VFA	0.15	0.10	0.25	1.55	0.15	2.75	0.75
HAc	0.12	0.09	0.18	1.16	0.11	1.70	0.56
HPr	0,04	0,03	0,20	1.94	0.21	2.55	0.3
pH value	7.72	7.70	8,00	7.99	7.87	8.08	7.70

[1] $ComCP_{min}$ and $ComCP_{max}$ correspond to the community-level change points at the 5th and 95th quantiles of 500 bootstrap replicates.
